# Evaluation of S- and M-Proteins Expressed in *Escherichia coli* and HEK Cells for Serological Detection of Antibodies in Response to SARS-CoV-2 Infections and mRNA-Based Vaccinations

**DOI:** 10.3390/pathogens11121515

**Published:** 2022-12-10

**Authors:** Mandy Schwarze, Ji Luo, Alexandra Brakel, Andor Krizsan, Nicole Lakowa, Thomas Grünewald, Claudia Lehmann, Johannes Wolf, Stephan Borte, Sanja Milkovska-Stamenova, Jörg Gabert, Markus Scholz, Ralf Hoffmann

**Affiliations:** 1 Institute of Bioanalytical Chemistry, Faculty of Chemistry and Mineralogy, Universität Leipzig, 04103 Leipzig, Germany; 2 Center for Biotechnology and Biomedicine, Universität Leipzig, 04103 Leipzig, Germany; 3 Adversis Pharma GmbH, 04103 Leipzig, Germany; 4 Klinik für Infektions- und Tropenmedizin, Klinikum Chemnitz gGmbH, 09113 Chemnitz, Germany; 5 Laboratory for Transplantation Immunology, Institute for Transfusion Medicine, University Hospital Leipzig, 04103 Leipzig, Germany; 6 Department of Laboratory Medicine, Hospital St. Georg gGmbH, 04129 Leipzig, Germany; 7 Immuno Deficiency Center Leipzig, Jeffrey Modell Diagnostic and Research Center for Primary Immunodeficiency Diseases, Hospital St. Georg gGmbH, 04129 Leipzig, Germany; 8 Institute for Medical Informatics, Statistics and Epidemiology, Universität Leipzig, 04107 Leipzig, Germany; 9 LIFE Research Center of Civilization Diseases, Universität Leipzig, 04103 Leipzig, Germany

**Keywords:** ELISA, epitope, immunoglobulin A (IgA), immunoglobulin G (IgG), peptide mapping, SARS-CoV-2

## Abstract

This study investigated the IgG and IgA antibody response against recombinant S1 and receptor binding domains (RBD) of the spike (S-) protein and the membrane (M-) protein using a set of 115 serum samples collected from patients infected with SARS-CoV-2 in Germany before April 2021 using protein and peptide ELISA. As S1- and RBD-proteins expressed in *Escherichia coli* provided poor sensitivities in ELISA, they were replaced by proteins expressed in HEK cells. The RBD-ELISA provided a sensitivity of 90.6% (N = 85) for samples collected from patients with confirmed SARS-CoV-2 infections more than 14 days after symptom onset or a positive PCR test. In population-based controls, the specificity was 97.9% (N = 94). In contrast, the sensitivities were only 41.2% and 72.6% for M- and N-proteins, respectively, while the specificities were 88.5% and 100%, respectively. Considering also 20 samples collected during the first two weeks of symptom onset or PCR confirmation, the sensitivity of RBD- and N-protein ELISA decreased to 82.6% and 72.6%, respectively. The combination of two data sets, i.e., N- and RBD-, N- and M-, or RBD- and M-proteins increased the sensitivity to 85.8%, 77.9%, and 87.8%, respectively. Peptide mapping mostly confirmed epitopes previously reported for S1- and M-proteins, but they were only recognized by a few samples already tested positive in the corresponding protein ELISA indicating that peptide-based assays will not improve the diagnostic sensitivity.

## 1. Introduction

The severe acute respiratory syndrome coronavirus 2 (SARS-CoV-2) pandemic causing coronavirus disease 2019 (COVID-19) is now in its third year [1,2]. It is still considered a global health threat with new virus variants of concern (VOCs) appearing every few months capable of infecting both vaccinated people and people recovered from previous SARS-CoV-2 infections. In July 2022, SARS-CoV-2 infections have been confirmed in more than 562 million persons including 6.3 million lethal cases [3]. Thus, it is still important to develop more accurate screening tools to identify infected individuals and to confirm the infection status retrospectively to judge future health threats, especially for newly emerging VOCs.

The genome sequence of SARS-CoV-2, which belongs to the *coronaviridae* [1], shows 79.5% and 50% identities to SARS-CoV and MERS-CoV, respectively. The single-stranded positive-sense RNA (+ssRNA) encodes four structural proteins [4,5], i.e., spike (S-), envelop (E-), membrane (M-), and nucleocapsid (N-) proteins. The S-protein consists of two subunits, that is the N-terminal S1 containing the receptor-binding domain (RBD), which initiates cell infection by binding to the angiotensin converting enzyme-2 (ACE-2) receptor, while the S2 subunit resembles the fusion domain [6,7]. The N-protein forms the ribonucleoprotein core, which is important for packaging, transcription, and replication of viral RNA [8]. The M-protein consists of three transmembrane structural domains and is the most abundant structural protein. It interacts with the N-protein to facilitate the molecular assembly of viral particles [9]. The E-protein, which is the smallest structural protein, plays a critical role in pathogenesis, viral assembly, and release [10]. 

Most laboratories rely on commercial serological assays detecting anti-SARS-CoV-2 antibodies on S- and N-proteins. However, varied sensitivity and specificity were observed [11,12,13,14,15,16,17] and the sources of recombinantly expressed proteins were often not mentioned or the methods were not reported in detail. Here, we explored the potential of an in-house M-protein-based ELISA in comparison to in-house RBD- and N-protein-based ELISA for serological testing of SARS-CoV-2 infections. In addition, we compared for all proteins the assay sensitivity and specificity when expressed in *E. coli* or HEK cells. The evaluation of different SARS-CoV-2 proteins and expression systems will facilitate adopting in-house assays without the need to buy expensive commercial kits. The study relied on 115 samples collected from hospitalized patients with PCR-confirmed SARS-CoV-2 infections in 2020 and early 2021, which allowed a fair comparison of the diagnostic value of M-, N-, and RBD-based assays using recombinant proteins. 

## 2. Materials and Methods

Reagents were obtained from the following manufacturers: Advansta Corporation (San Jose, CA, USA): WesternBright Sirius^®^; Biosolve BV (Valkenswaard, The Netherlands): Dimethylformamide (DMF, peptide synthesis grade), piperidine (≥99.5%); Carl Roth GmbH & Co. KG (Karlsruhe, Germany): Carbenicillin disodium salt, lysogeny-broth (LB) medium, lysozyme (≥45,000 FIP U/mg), ROTI^®^Stock 10× PBS, ROTI^®^Stock 10× PBS-T, sodium chloride (≥99.5%), sodium dodecyl sulfate (SDS, ≥99.5%), sulfuric acid, terrific-broth (TB) medium, thioanisole (≥99%) and urea (>99.5%); Honeywell Fluka^TM^ (Seelze, Germany): Ethane-1,2-dithiol; Promega GmbH (Mannheim, Germany): Peroxidase-conjugated anti-human IgG antibody; Roche Deutschland Holding GmbH (Mannheim, Germany): cOmplete™ Mini EDTA-free protease inhibitor cocktail (from bovine pancreas); Seramun Diagnostika GmbH (Heidesee, Germany): TMB substrate solution; SERVA Electrophoresis GmbH (Heidelberg, Germany): Acrylamide/bis(acrylamide) (30% T, 2.67% C), ammonium persulfate (APS; analytical grade), BlueBlock PF 10×, Coomassie Brilliant Blue G-250, *N*,*N*,*N*′,*N*′-tetramethylethane-1,2-diamine (TEMED, analytical grade), and trypsin (sequencing grade, MS approved); Sigma Aldrich Chemie GmbH (Taufkirchen, Germany): 2-mercaptoethanol (BioUltra), Antifoam Y-30 emulsion, hydroxybenzotriazole (HOBt, ≥97%), imidazole (≥99.5%), *m*-cresol (≥99%), *N*,*N*′-diisopropylcarbodiimide (DIC, ≥98%), polyethylenimin (PEI), and trifluoroacetic acid (TFA, for HPLC, >99%); Surmodics IVD, Inc. (Eden Prairie, MN, USA): StabilZyme^TM^ SELECT; Thermo Fisher Scientific (Waltham, MA, USA): SuperBlock^®^ (PBS), Gibco DMEM, Gibco Fetal Bovine Serum (FBS), Gibco GlutaMAX figure ement, Gibco 100x MEM non-essential amino acids solution, goat anti-human IgA secondary antibody-HRP, and penicillin/streptomycin (10,000 U/mL); VWR International GmbH (Darmstadt, Germany): Acetonitrile (HPLC-gradient grade), diethyl ether, and formic acid (98%).

### 2.1. Serum Collection

Clinical serum samples were obtained from patients hospitalized in 2020 (N = 113) and 2021 (N = 2) with SARS-CoV-2 infections confirmed by PCR (Hospital St. Georg, Leipzig, Germany and Klinikum Chemnitz, Chemnitz, Germany) including 49 samples lacking the information of symptom onset (Table S1). These investigations represent parts of the analyzes in the COVID genetics cohort Leipzig-Chemnitz, which was approved by the Institutional Review Board of Leipzig University (reference numbers 195/20-ek and EK-allg-37/10-1). Control serum samples collected throughout the year from 2009 to 2015 and considered to be negative for SARS-CoV-1/2 and MERS-CoV infections were obtained from the population-based LIFE-Adult study of the Leipzig Research Center for Civilization Disease (LIFE) [18,19]. Additionally, serum samples (N = 84) were collected from 21 non-infected persons vaccinated with mRNA-based vaccines BNT162b2 and/or mRNA-1273 and five vaccinated infected individuals from May 2021 to May 2022 (Table S2).

### 2.2. Recombinant Proteins Expressed in E. coli

The coding sequence of S1- (C-terminal His_6_-Tag, residues 1-711, NCBI accession YP_00972439), RBD (C-terminal His_6_-Tag, residues 319–541, NCBI accession YP_00972439) and the M-protein (N-terminal His_6_-Tag, NCBI accession # QHR63253.1) were synthesized and codon-optimized for *E. coli* by GenScript Biotech BV (Leiden, The Netherlands) and cloned into pET23a(+), pET21b(+), and pET28a(+) vectors, respectively. The vectors were transformed into *E. coli* Rosetta(DE3) or BL21 pLysS. Single colonies of *E. coli* Rosetta(DE3) pET28a(+)-M-protein, Rosetta(DE3) pET28a(+)-S1-protein and BL21 pLysS pET21b(+)-RBD were cultivated in LB and TB medium containing kanamycin (50 mg/L) or ampicillin (10 mg/L) on a horizontal shaker (200 rpm, Certomat^®^ BS-T, B. Braun Biotech GmbH, Melsungen, Germany) at 30 °C (M-protein) or 37 °C (S1- and RBD-proteins). Protein expression was induced by addition of IPTG (1 mmol/L) when the OD_600_ of the bacterial culture reached 0.4 to 0.6. Cells were incubated under constant shaking (200 rpm) at 30 °C for 16 h (M-protein) or 37 °C for 4 h (S1 and RBD), harvested, and resuspended in PBS containing lysozyme (50 µmol/L) and a protease-inhibitor mix (1 tablet/10mL). Cells were disrupted on a French^®^ Press (Thermo Fisher Scientific, Waltham, USA) or FastPrep-24 5G (MP Biomedicals, Eschwege, Germany) and centrifuged (48,000× *g*, 4 °C, 1 h). The pellet was suspended in high-salt PBS (35 mL), i.e., PBS supplemented with NaCl (350 mmol/L) and centrifuged (30 min, 4 °C). The suspension was repeated with the same volumes of high salt-PBS containing Triton X-100 (1%, *v*/*v*), high-salt PBS containing urea (2 mol/L), and high-salt PBS containing urea (4 mol/L) followed each time by centrifugation (30 min, 4 °C). The pellet containing the inclusion bodies was dissolved in high-salt PBS (30 mL) containing urea (8 mol/L). Proteins were purified by immobilized metal ion affinity chromatography (IMAC) using a HisTrap^TM^ HP column (GE Healthcare, Chicago, IL, USA). The N protein was expressed as previously described [17]. 

### 2.3. Recombinant Proteins Expressed in HEK Cells

A commercial pUC vector harboring the sequence of the S-protein (GenScript Biotech BV, Leiden, The Netherlands) was used to clone the sequences of RBD (318–541 bp), S1 (13–1273 bp), and full-length M-protein (1–222 bp) into expression vector pHLsec together with a C-terminal His-tag (RBD-protein) or C-terminal TwinStrep-Tag and N-terminal His-Tag (S1- and M-proteins). Transient transfection of HEK293S cells with the RBD and M-protein expression vector, was achieved by incubating the plasmid vector (3 mg) and PEI (4.5 mg) in DMEM (150 mL) containing MEM non-essential amino acids solution (10 mmol/L) and GlutaMAX (2 mmol/L) at room temperature (RT) for 15 min. A stable cell line was established for the S1-protein using the PiggyBac system, as described in the manufacturer’s manual [20]. Briefly, the insert of the cloned pHLsec vector (C-terminal His-tag) was transferred into the PiggyBac vector and a stable cell line was generated under puromycin selection. The successful insertion of the constructs into the genome of HEK cells was monitored by co-expressing the green fluorescent protein (GFP). The transfection mix (25 mL, transient expression) and penicillin/streptomycin solution (2.5 mL) were added to HEK cells grown in DMEM (225 mL) containing FBS (10%, *v*/*v*), MEM non-essential amino acids solution (10 mmol/L), and GlutaMAX (2 mmol/L) in a RollerBottle (Greiner Bio-One, Frickenhausen, Germany) at 37 °C. The RBD-protein was harvested after five days and the S1-protein after ten days. The proteins were purified by IMAC using a HisTrap^TM^ HP (GE Healthcare) column (RBD-protein) and StrepTactin affinity chromatography using a StrepTrap^TM^ HP (Cytiva, Marlborough, MA, USA) column (S1-protein, M-protein) following the manufacturers’ guidelines. Size-exclusion chromatography (SEC) followed for the proteins (RBD, S1), as recently reported [21]. 

### 2.4. Peptide Synthesis

Peptides were synthesized by Fmoc/*^t^*Bu-chemistry on Rink amide or Wang resin as C-terminal amides or free acids, respectively, on the multiple synthesizer SYRO 2000 (MultiSynTech GmbH, Witten, Germany). Protected amino acids were activated *in situ* with DIC/HOBt. Peptides were cleaved with TFA containing 12.5% (*v*/*v*) of scavenger mixture (ethane-1,2-dithiole, *m*-cresol, thioanisol, and water, 1:2:2:2, (*v*/*v*)) and precipitated with ice-cold diethyl ether, and dried. Peptides were purified by RP-HPLC using a linear acetonitrile gradient in the presence of 0.1% TFA. Peptide masses were confirmed by ESI-MS and the purities were determined by RP-HPLC recording the absorbance at 214 nm.

### 2.5. Enzyme-Linked Immunosorbent Assay (ELISA)

The ELISA was optimized for each protein, as described in the Figures S1–S4. Medium binding microplates (Greiner Bio-One, 12×F8, PS, F-bottom) were coated in each well with RBD- or S1-proteins expressed in HEK (75 ng) in PBS supplemented with sodium chloride (200 mmol/L) or RBD- (150 ng) and S1-proteins (250 ng) expressed in *E. coli* in PBS at 4 °C overnight. The M-protein (125 ng/well) was coated in PBS containing urea (4 mol/L) at 4 °C overnight. Wells were washed three times with PBS-T (pH 7.4, 300 µL) and blocked with Superblock (200 µL, RT, 1 h). The positive control consisted of 18 pooled samples from patients with confirmed SARS-CoV-2 infections used. The negative controls of the M-protein and RBD-/S1-protein ELISA were prepared by pooling serum samples of the LIFE-Adult collection, respectively. All samples were diluted 100-fold in assay diluent and incubated at RT for 45 min. Wells were washed with PBS-T (300 µL/well) before anti-human IgG-HRP (100 µL/well) was added as a 30,000-fold (M-protein) or 25,000-fold dilution (RBD- and S1-proteins) in Stabilzyme. Alternatively, anti-human IgA-HRP (100 µL/well) was added as a 10,000-fold dilution in Stabilzyme. After 30 min at RT, wells were washed three times with PBS-T (pH 7.4, 300 µL/well) before TMB substrate solution was added (100 µL/well). The reaction was stopped after 10 min by the addition of sulfuric acid (0.5 mol/L; 100 µL/well) and the absorbance was recorded at 450 nm using a microplate reader (SUNRISE, Tecan Group AG, Männedorf, Switzerland or SpectraMax Paradigm, Molecular Devices, München, Germany). The cutoff value was determined by Receiver Operating Characteristics (ROC) analysis to obtain the best sensitivity and specificity based on a maximal Youden index using GraphPad Prism versions 9.0.2 or 5.01 (Graph Pad Software, La Jolla, CA, USA). N-protein ELISA was performed as previously described [17].

### 2.6. Peptide ELISA

Aqueous peptide solutions (10 mg/L; 100 µL/well) were coated on Maxisorp plates without cover to dry the solutions (37 °C, 20 h). All following steps were performed at room temperature. Wells were blocked with Superblock^TM^ (200 µL/well) for at least 60 min. Serum was added (100 µL/well, 100-fold diluted in Stabilzyme or PBS-T) and the plate was incubated for 45 min. The wells were washed three times with PBS-T. IgG conjugate (100 µL/well, 20,000-fold diluted in Stabilzyme or PBST) was added to each well and incubated for 30 min. The plates were washed three times with PBS-T (300 µL/well) and TMB substrate solution was added (100 µL/well). After 10 min, the reaction was stopped by adding sulfuric acid (0.5 mol/L, 100 µL/well) and the absorbance was recorded at 450 nm using a SUNRISE or SpectraMax Paradigm microplate reader.

## 3. Results

### 3.1. Expression and Purification of Recombinant SARS-CoV-2 Proteins

The SARS-CoV-2 S1- and RBD-proteins were successfully expressed in both *E. coli* Rosetta(DE3) and HEK293S cells and purified by IMAC, as indicated by strong single bands in SDS-PAGE after reduction, which was also the case for the *E. coli* M-protein after reduction and alkylation (Figure S5). The RBD- and S1-proteins expressed in *E. coli* were detected at the expected apparent molecular weights of ~26 kDa and ~78 kDa, respectively, while the corresponding proteins expressed in HEK cells appeared at higher molecular weights indicating that both proteins were glycosylated. Interestingly, IMAC separated the crude RBD-protein preparation in a main fraction containing monomeric RBD and an earlier eluting fraction consisting of dimeric RBD, which was confirmed by non-reducing SDS-PAGE. The protein identities were confirmed by immunoblotting using an anti-His antibody and LC-MS after in-gel digestion.

In the case of the *E. coli* M-protein, a double band was seen in the gel where the monomeric protein was expected. In contrast, the M-protein expressed in HEK cells migrated as a single band in this part of the gel, which corresponded to the upper band seen for *E. coli* M-protein. Since only a single glycosylation site is predicted for the M-protein, the expected mass shift of ~1.2 kDa (HEK S cells) may have only a minor effect on the migration of the M-protein. The SDS-PAGE displayed several more bands above the expected apparent molecular weight, which were all recognized in an immunoblot by an anti-His antibody (*E. coli*) or Strep-HRP conjugate (HEK). When the gel bands were cut-out, digested with trypsin, and analyzed by LC-MS, the M-protein was detected in all these bands. Most likely, the M-proteins formed oligomers that were even stable during SDS-PAGE despite reducing and denaturing conditions (Figure S5).

### 3.2. ELISA

**Figure 1:**
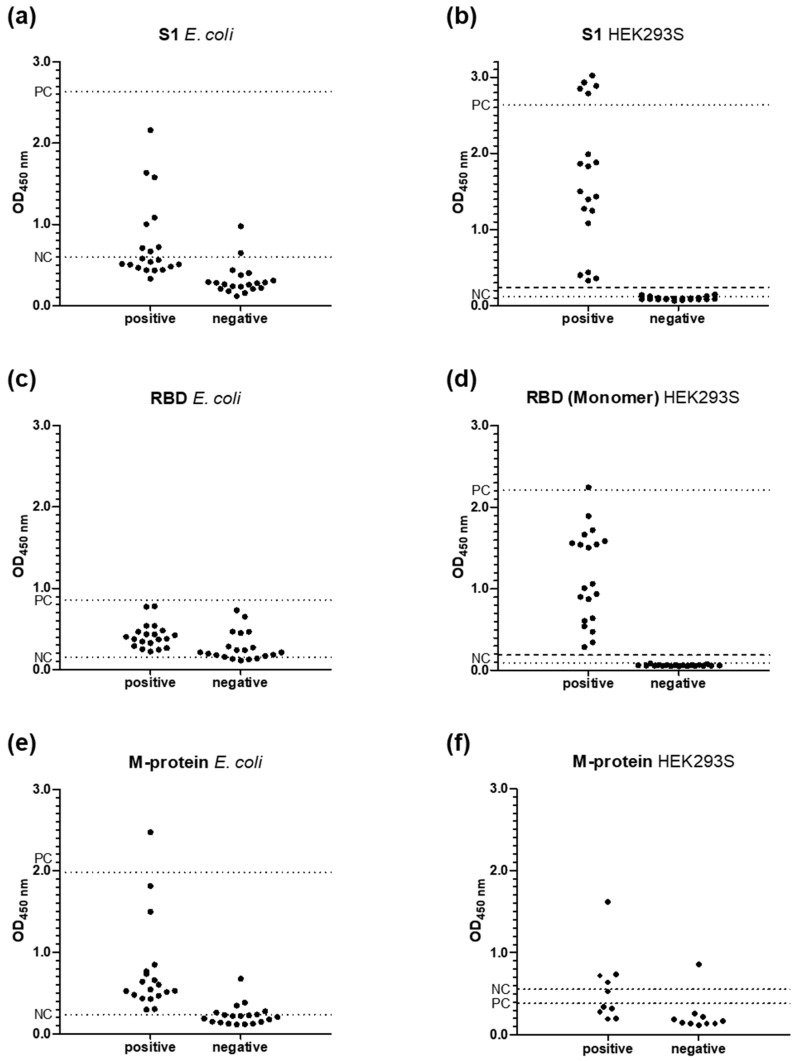
Distribution of OD_450_-values obtained by testing 20 SARS-CoV-2 positive and 20 negative human serum samples in ELISA for IgG antibodies recognizing recombinant proteins S1 (**a**,**c**) and RBD (**b**,**d**) expressed in *E. coli* (**a**,**b**) or HEK cells (**c**,**d**). The M-protein expressed in *E. coli* (**e**) or HEK cells (**f**) was tested against the same set of 40 samples or a subset of ten positive and ten negative samples, respectively, due to the extremely low expression rates in HEK cells. OD_450_-values measured for positive (PC) and negative (NC) serum pools on each plate are indicated as dotted lines, which used in the following assays for normalization (i.e., PC = 100%, NC = 0%). The cutoff values calculated for S1- (0.24, **b**) and monomeric RBD-proteins (0.19, **d**) expressed in HEK cells are indicated by dashed lines.

The purified RBD-, S1-, and M-proteins expressed in *E. coli* or HEK293S cells were tested in ELISA against one set of SARS-CoV-2 positive (N = 20) and negative samples of the LIFE-Adult group (controls, N = 20) with respect to assay sensitivity and selectivity by detecting human IgG antibodies (Figure 1). RBD- and S1-proteins expressed in *E. coli* did not provide a good separation of positive and negative sera with the corresponding ROC curves providing a sensitivity of only 95.2% and even worse specificities of 66.7% and 81%, respectively, while the corresponding proteins expressed in HEK cells, including dimeric RBD, all provided sensitivities and specificities of 100% (Figure S6). The OD_450_ values of the positive samples ranged from 0.33 to 3.02 for S1 and from 0.29 to 2.24 for RBD, which were well separated from the negative controls ranging from 0.06 to 0.14 and 0.06 to 0.09, respectively. The cutoff values of RBD and S1 were 0.19 and 0.24. M-protein expressed in *E. coli* provided a better separation of negative and positive sera than both S1- and RBD-protein preparations expressed in *E. coli* with OD_450_-values ranging from 0.12 to around 0.4 and from 0.3 to 2.47, respectively. A ROC curve analysis provided an excellent sensitivity of 100%, but only a moderate specificity of 85%.

The RBD- and S1-proteins expressed in HEK cells provided very similar results and thus the RBD appeared to be more appropriate for ELISA, as the expression rate was 5-fold higher and as it represents the domain initiating cellular infection by binding to the human ACE2 receptor [22,23]. Many companies offer RBD-based serological test kits, such as ACE2 Inhibitor Screening Assay Kit (BPS Bioscience, San Diego, California), Angiotensin I EIA Kit (Merck KGaA, Darmstadt, Germany), and SARS-CoV-2 S1RBD/ACE2 Binding Assay Kit (BIOZOL Diagnostica Vertriebs GmbH, Eching, Germany). As *E. coli* RBD did not provide good results in the IgG-based ELISA, although it bound to the ACE2 receptor, it was not further considered for testing [24]. Thus, RBD was expressed in HEK cells, as previous reports indicated also a good sensitivity in ELISA [16,25,26,27]. When the monomeric and dimeric versions of RBD expressed in HEK cells in-house and two commercial RBDs were coated in ELISA, the obtained OD_450_-values were identical within the error range (Figure S7). Only the readouts of the positive sera were slightly higher for the RBD dimer, most likely due to an improved coating. As the monomeric RBD was obtained in much higher yields than the dimeric RBD and provided very similar results, it was used for further ELISA testing.

M-proteins expressed in *E. coli* and HEK cells provided similar results (Figure 1). The negative pool recognized the HEK cell-derived M-protein even better than the positive pool, suggesting a strong cross-reactivity to other virus proteins. Thus, the M-protein expressed in *E. coli* was used for further studies due to the much higher expression rates.

### 3.3. RBD-Protein ELISA

**Figure 2:**
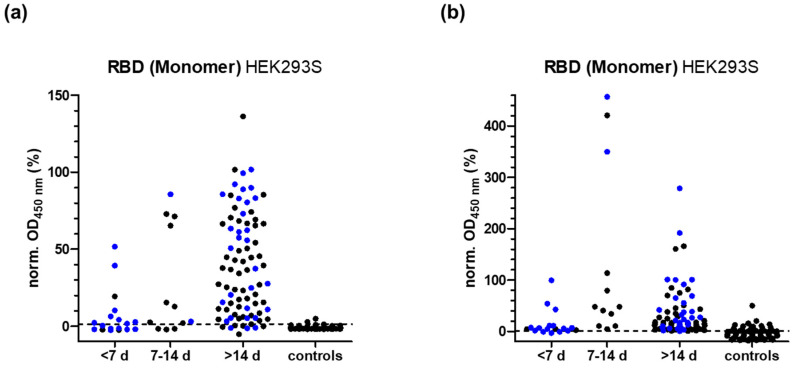
Distribution of normalized OD_450_-values measured in ELISA for all SARS-CoV-2 positive samples of groups 1 to 3 (N = 115) and 94 control human serum samples by coating RBD-protein expressed in HEK cells and probing for IgG (**a**) or IgA antibodies (**b**). Samples were grouped based on the time period after symptom onset (black dots, N = 67) or a positive PCR test (blue dots, N = 48). The OD_450_-values determined for positive (set to 100%) and negative pools (set to 0%) on each plate were used for normalization. The cutoff values calculated for IgG (1.25%, **a**) and IgA (0.52%, **b**) are indicated as dashed lines.

Based on the period between symptom onset and sample collection, the positive serum samples (N = 67) were divided in three groups: less than 7 days (N = 3, group 1), 7 to 14 days (N = 10, group 2), and more than 14 days (N = 54, group 3). Additionally, 48 samples with unknown symptom onset were attributed to the groups based on the time period between PCR confirmation and sample collection (N = 115 in total), i.e., 15 samples to group 1 (N = 18 in total), two samples to group 2 (N = 12), and 31 samples to group 3 (N = 85) (Table S1). The control group consisted of 94 samples randomly chosen from the LIFE-Adult group. The ROC curve analysis provided for the RBD-protein ELISA probed for IgG antibodies a sensitivity of ~83.8% and a specificity of ~97.9% with a cutoff of 1.25% (Figure S6). The low sensitivity was mostly attributable to samples collected in the early phase of infection, before a sufficient immune response can be expected, as the sensitivity increased from 50% in group 1 to 75% in group 2, and to 90.6% in group 3 (Table 1, Figure 2). 

When the same RBD-protein ELISA was used to test for IgA antibodies (Figure 2b), the sensitivity increased especially for the earlier periods from 50.0% to 83.3% in group 1, from 75% to 100% in group 2, and from 90.8% to 98.8% in group 3 (Table 1). However, the value of this sensitivity is limited by the low specificity of only 84.0%.

**Figure 3:**
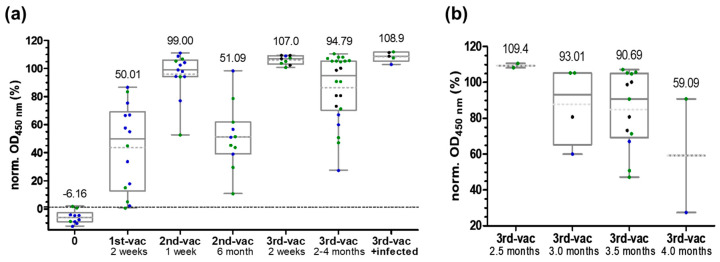
Box plots of normalized OD_450_-values obtained for testing serum samples collected from individuals vaccinated with mRNA vaccines BNT162b2 (green), mRNA-1273 (blue) or mix of both (black) in the RBD-protein ELISA probed for IgG. Panel (**a**) shows the whole period, while panel (**b**) shows in detail the data for samples collected two to four months after the third vaccination. Normalization relied on OD_450_-values of positive (PC) and negative pools (NC) set to 100% and 0%, respectively. The cutoff (1.25%) is indicated as a dotted line.

An important application of the RBD-protein ELISA is the investigation of the vaccination status, because most antibodies recognizing the RBD will most likely reduce or abolish the binding to ACE-2, as confirmed by neutralizing assays [21]. The current study focused on mRNA vaccines BNT162b2 and mRNA-1273 in different combinations and monitoring the immune response for up to three vaccinations (Table S2). Expectedly, all sera collected before the first vaccination were negative with a median of -6.2% (normalized OD_450_ values below 1.25%, Figure 3a). 

The OD values increased for most individuals after the first vaccination to a median of 50%, but with a very high variation among persons ranging from 0.6% to 86.7%. The second vaccination increased the values to a median of 99.0%, with only two sera samples below 80%. Interestingly, these values were already higher than for infected hospitalized persons two weeks after symptom onset or a positive PCR test. However, the levels decreased in vaccinated people over the following six months to 51.1% independent of the vaccine, again with a large variation among individual persons ranging from 10.8% to 98.4%. Two weeks after the third vaccination, an even higher median of 107.0% with a very low variation among individuals was observed confirming the effectiveness of a third mRNA vaccination. In the following two to four months, the levels decreased for some persons only slightly, but rapidly for others to a minimum of 27.4% (median: 94.8%, Figure 3b). Considering different time periods, the median decreased slowly in the two-month observation period. Although the low number of samples do not allow clear conclusions, it evidently suggests a person-dependent development of the antibody titer. It should be noted that none of the persons were tested positive for SARS-CoV-2 during the observation period by regular COVID-19-antigen tests (nasal swab). This was further confirmed by negative anti-N-protein ELISA with only one serum in the borderline range (Figure S8). Five individuals enrolled in the vaccination study were infected with SARS-CoV-2 around three to five months after the third vaccination, most likely with an omicron variant. Afterwards, the OD values of all persons were above 100% with a median of 108.9%, which was even higher than after the third vaccination (Figure 3a). As expected, the serum samples collected post-infection were tested positive in the N-protein ELISA, while all previously collected samples were negative.

### 3.4. S- and RBD-Peptide ELISA

Peptides reported to be immunogenic [22,28,29] were tested against sera of infected and non-infected persons for IgG titers (Table S3). On average, the absorbance was 0.73 for the positive pool and 0.29 for the negative pool indicating a high background resulting in a low sensitivity for all tested peptides (Figure S9a). Even the most promising peptides S22 and S803 tested in a second round against 30 sera from infected patients and 47 control sera (Figure S9b) could not separate both groups and provided only low specificity and selectivity. Further optimization of the ELISA conditions, e.g., altered coating and blocking conditions as well as solutions used to dilute sera and antibodies, did not satisfactorily improve the results. Therefore, the S- and RBD-peptide ELISA was not further pursued.

### 3.5. M-Protein ELISA

**Figure 4:**
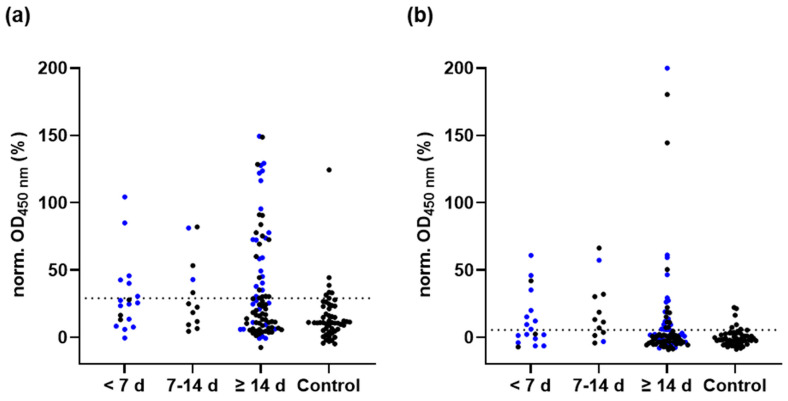
Normalized OD_450_-values of the M-protein ELISA probed for IgG (**a**) and IgA (**b**) antibodies using all serum samples of groups 1 to 3 based on symptom onset (black dots, N = 48) or PCR test confirmation (blue dots, N = 67) and 52 control samples. The OD_450_-values determined for positive (= 100%) and negative pools (= 0%) on each plate were used for normalization. The cutoff values of the IgG (29.10%, **a**) and IgA tests (5.55%, **b**), based on ROC curve analyses, are indicated as dashed lines.

While S- and N-protein ELISA showed a good sensitivity for SARS-CoV-2 infected persons, there were still many samples from infected persons that were not detected by either of these serological tests. Thus, we tested also the M-protein for its diagnostic suitability and if such an assay could close the gap of established ELISA, especially for identifying past infections of persons previously vaccinated with S-protein based vaccines. When 115 sera collected within the first six days after symptom onset or PCR (group 1, N = 18), from day 7 to 13 (group 2, N = 12) and on day 14 or later (group 3, N = 85), were tested for anti-M-protein IgG antibodies, the M-protein ELISA correctly identified six sera of group 1 (33.3%), five sera of group 2 (41.7%), and 35 sera of PCR group 3 (41.2%) as positive (Figure 4a). Interestingly, when tested for IgA antibodies, the sensitivity increased to 50.0% in group 1 and to 66.7% in group 2, but fell to only 31.8% in group 3 (Figure 4b). As two of the nine samples tested positive for IgG were negative for IgA, the sensitivity based on both antibodies was 61.1% for group 1 (Figure S10a). Similarly, the seven samples of group 2 tested only IgA-positive, which increased the overall sensitivity to 75.0% (Figure S10b). 

Six serum samples of the control group (N = 52) were falsely tested positive for anti-M-protein IgG or IgA, which relates in both cases to a specificity of 88.5%, including two samples tested positive for both anti-M-protein IgG and IgA. As all control samples were negative in the RBD-protein ELISA and a N-protein ELISA, the false positive samples may indicate contaminating *E. coli* proteins, which was not further investigated.

### 3.6. M-Peptide ELISA

Based on recently reported epitope regions 1–20 and 201–222 of the M-protein for COVID-19 patients [30], we tested a total of six peptides corresponding mostly to the N-terminal region 1 to 21, the C-terminal region 173 to 222, and residues 101 to 113 synthesized in-house (Table S3). The most interesting were peptides M4 (residues 4 to 21) and M204 (residues 204 to 222) (Figure S11). The M4 peptide correctly identified six sera of group 1 (33.3%), four sera of group 2 (33.3%), and 33 sera of group 3 (39.1%) as positive (Figure S12a), while the M204 peptide identified three (16.7%), three (25%), and 38 sera (44.8%) as positive, respectively (Figure S12b). While most positive sera recognized also the full-length M-protein, peptide M4 additionally identified one serum each in groups 1 and 2 (Figure S13a) and peptide M204 additionally four samples in groups 3 (Figure S13b). The specificity of M4 and M204 peptide ELISA were 84.6% and 86.5% (N = 52), respectively. Three control samples incorrectly identified in the M-protein IgG ELISA were also false-positive in both peptide ELISA. Considering that the M-peptide ELISA correctly identified only six additional sera as positive, but with reduced specificity, we did not further investigate this peptide ELISA. 

### 3.7. False Positive Results in N- and RBD-Protein ELISA

In addition to the samples mentioned above, the RBD-protein based ELISA correctly identified 28 samples collected from non-infected persons before 2015 as negative, including 18 samples that were incorrectly identified as positive (false positives) in our previously reported N-protein based ELISA (Figure S14) [17]. However, the RBD-protein ELISA incorrectly identified two of the above mentioned 94 control sera as positive, although these samples were correctly identified as negative in the N-ELISA (Figure S14).

## 4. Discussion

Several companies have developed N- and S-protein-based ELISA for serological testing of previous SARS-CoV-2 infections with reported sensitivities ranging from 53.7% to 93.1% and from 77.1% to 89.2%, respectively. This variation likely reflects differences in the reagents, protein expression systems, protein purity, and protocols used for these ELISA and most likely also the cohort of clinical samples from patients with different etiopathologies. The success of recent S-protein-based vaccination campaigns has led to high anti-S-protein antibody titers in the general population, leaving in vaccinated persons only N-protein-based ELISA for the detection of past SARS-CoV-2 infections. S-protein-based ELISA are used to control antibody titers in vaccinated persons or recovered patients, which allows also judging neutralizing antibody titers. However, the SARS-CoV-2 virus contains further immunogenic structural proteins that can induce an immune response allowing serological assays to screen for past infections [1,31,32], such as the M-protein. The M-protein is the most abundant protein in the virus particle and induces both humoral and cellular immune responses [33], but it is rarely used in serological tests, which typically use N- or S-proteins.

The current study intentionally relied on serum samples obtained from patients infected with a SARS-CoV-2 variant in Germany before April 2021 before variants of concern containing several escape mutations were identified, and persons vaccinated with a mRNA vaccine without being infected by SARS-CoV-2 prior to sample collection. Thus, it was possible to evaluate the production of antibodies against different SARS-CoV-2 proteins relying on the sequences of the SARS-CoV-2 variant emerging in Europe, which was very close to the original Wuhan strain, to judge the sensitivity of assays by combining different viral proteins, and to compare the immune response against the S-protein in infected and vaccinated persons. Despite high purities, S1- and RBD-proteins expressed in *E. coli* provided only low sensitivities and specificities and were thus not considered for further assay development. These proteins were likely incorrectly folded presenting epitopes buried in native proteins or masked by glycosylation sites, although recognition of contaminating *E. coli* proteins cannot be excluded [34]. Glycosylation of the RBD-, i.e., known glycosylation sites at Thr323, Ser325, Asn331, and Asn343, and S-proteins expressed in HEK cells were confirmed by their slower migration in SDS-PAGE compared to the corresponding proteins expressed in *E. coli* and a glycostain (Figure S15). In addition, glycosylation was confirmed in the RBD using mass spectrometry after tryptic digestion (manuscript in preparation). Similar to *E. coli* proteins, linear epitopes identified by peptides might be useful for diagnostic purposes. However, all peptides corresponding to the S-protein including two RBD-peptides tested here, were recognized only by a small subset of samples, which could not be linked to disease severity. As both proteins expressed in HEK cells were equally well recognized by the same serum samples, the RBD-protein was preferred due to its better expression rates. More importantly, the RBD-protein represents the ACE-2 binding site and thus antibodies recognizing this protein in ELISA will probably also inhibit RBD-ACE-2 interactions. Thus, the ELISA will also indicate neutralizing effects, although advanced neutralization assays will provide more reliable data [21,35,36].

Almost all commercial serological tests probing anti-SARS-CoV-2 antibodies rely on S- and N-proteins. Assuming that the M-protein might be useful to identify serum samples collected from SARS-CoV-2 infected persons that were not identified in S- and N-protein-based tests, we explored an in-house full-length M-protein ELISA. When Lopandic et al. used an ELISA coated with a shortened, 149 residues long M-protein lacking transmembrane regions 20–103, the sensitivity was 96.7% (N = 30) and the specificity was 92.5% (N = 40) [37], which is close to the sensitivity of 90.0% (N = 20) and specificity of 95% (N = 20) we observed for the initial small sample set for test optimization. However, these parameters changed in the large serum sample set, the sensitivity dropped to only 41.2% in group 3 (N = 85) and the specificity fell slightly to 88.5% (N = 52). These uncommonly high differences in sensitivity might be linked to a ‘lucky random’ serum selection of the small data set. The N-terminal (aa 1–20) and C-terminal (aa 202–222) epitopes previously identified in 20.0% and 10.0%, respectively, of plasma samples of 30 convalescent of COVID-19 patients (28–84 days after symptom onset) [30], appear to be even more common based on our numbers. The recognition of M-peptides by some sera that did not detect the M-protein might indicate an improper folding of the M-protein expressed in *E. coli*. This may be further supported by the low overall sensitivity of the M-protein ELISA similar to the differences obtained for RBD- and S1-proteins expressed in either *E. coli* or HEK cells, especially as the full-length M-protein showed a strong aggregation. However, the M-protein expressed in HEK cells did also not improve the specificity. Even more surprising were the higher OD-values observed for the negative control compared to the positive control, which might indicate a cross-reactivity of antibodies produced in response to other corona viruses. This could be verified using a larger sample set. It is unlikely that the lack of glycosylation played an important role, since expression of a glycosylation-deficient SARS-CoV M-protein does neither influence the shape of the virions nor their infectivity in cell cultures [38].

The sensitivities of the RBD-protein ELISA ranged from 50% in the early stage to 90.8% in late stages of COVID-19, while the M-protein provided lower sensitivities of 33.3% to 41.7% considering IgG antibodies. The sensitivity of the RBD ELISA in group 3 was comparable to previously reported ELISA results using samples collected at least three weeks after diagnosis of SARS-CoV-2 [25,26]. Algaissi et al. also reported a sensitivity of 40–60% in the S1 IgG ELISA for earlier time points [16], which relates very well to the current study. The M-protein ELISA was worse in detecting positive sera than the RBD-protein ELISA independent of the *E. coli* and HEK cell expression systems, especially when considering its low specificity, which is most likely related to a cross-reactivity of antibodies induced by other (corona)viruses. 

**Figure 5:**
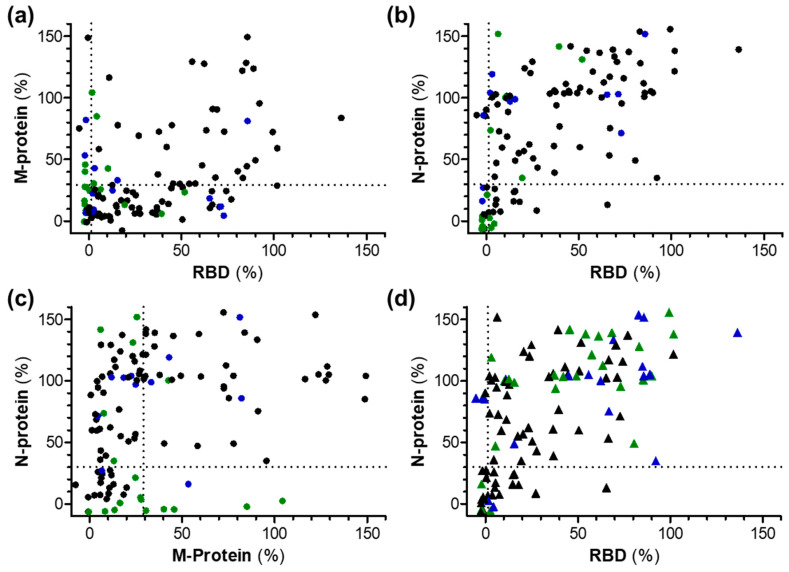
Scatter plots displaying the normalized OD_450_-values obtained for all serum samples. The ELISA results of groups 1 (green dots), 2 (blue dots), and 3 (black dots) are shown for combinations of RBD- (**a**,**b**), N- (**b**,**c**), and M-proteins (**a**,**c**) as well as for all three proteins (**d**) probed for IgG antibodies. Triangles indicate the normalized M-protein OD_450_-values below the cut-off of 29.1% (black), from 29.1 to 70% (green), and above 70% (blue) (**d**). The samples were normalized to the OD_450_-values of positive (=100%) and negative pools (=0%). Dotted lines indicate the cutoffs of the respective ELISA (RBD-protein: 1.25%, M-protein: 29.1%, and N-protein: 30%).

For a broader discussion of anti-SARS-CoV-2 antibodies, previously reported data of our N-protein ELISA were also considered here (Figure S16) [17]. Sera collected in early disease stages, i.e., samples in groups 1 and 2, recognized N-protein and M-proteins (N = 4 for both proteins), while the RBD-protein ELISA was negative (Figure 5). This could indicate that in early disease stages the immune system produces antibodies directed against different viral proteins, before more specific antibodies directed against one or two proteins are selected. Interestingly, the detection of IgA antibodies in the M-protein ELISA increased the sensitivity of this assay from 36.7% to 66.7% and from 82.6% to 94.7% in the RBD-protein ELISA considering all samples of groups 1 and 2.

As none of the assays identified all of the positive samples and several samples were only positive in RBD- or M-protein ELISA, we considered also combining data of both ELISA or the data of each ELISA with a previously reported N-protein ELISA [17], which provided a sensitivity of 89.7% when tested against the same set of samples. In case that the normalized OD-values were plotted for two proteins against each other, there were always several samples below the cutoff values of both proteins (Figure 5). Interestingly, the combination with the M-protein ELISA increased the sensitivities of the RBD- and N-protein ELISA from 82.6% (samples from group 1–3) and 72.6%, respectively, to 87.8% and 77.9%. The combination of RBD- and N-protein ELISA improved the sensitivity for the RBD from 82.6% to 85.8%, while the N-protein ELISA missed 15 samples identified in the RBD-protein ELISA. Additionally, the normalized OD-values obtained in the N-, M-, and RBD-protein ELISA showed very low correlation coefficients of 0.37, 0.15, and 0.09 for the combination of N- and RBD-, M- and RBD-, and M- and N-proteins, respectively. The combined sensitivities of M-/RBD-, N-/RBD-, and N-/M-protein ELISA were 87.8%, 85.8%, and 77.9% for all groups, respectively.

Interestingly, the 2.1% false positive rate of control sera using either N- or RBD-protein ELISA were negative in the other assay. The false positive rates are most likely related to a cross-reactivity of antibodies produced in response to another corona virus infection [29,34,39]. The combination of all three proteins provided a sensitivity of 88.7%, but this is only valid for non-vaccinated persons. Due to the current S-protein-based vaccination campaign, the N-/M-protein pair appears most interesting for epidemiological studies about infection rates in the general population.

## 5. Conclusions

This study investigated the immune response in patients hospitalized after an infection with a SARS-CoV-2 variant in 2020 using recombinant S1-, RBD-, N-, and M-proteins in established in-house ELISA. S1- and RBD-proteins expressed in *E. coli* provided only low sensitivities and specificities. As both proteins expressed in HEK cells were recognized in ELISA by the same serum samples, the RBD-protein was used for further testing. When serum samples collected at least 14 days after symptom onset or positive PCR were tested for IgG antibodies, the sensitivity of the RBD-protein ELISA was 90.8% and the specificity was 97.9% based on a set of 94 control samples. Using the same set of samples, the sensitivities of M- and N-protein ELISA were 41.2% and 72.6%, while the specificities were 88.5% and 100%. When the samples were tested for both IgG and IgA response, the sensitivities increased, especially for serum samples collected within the first weeks after symptom onset or PCR confirmation. When all positive results of two different ELISA probed for IgG antibodies were combined, the combination of N-/RBD-, N-/M-, and RBD-/M-protein ELISA provided increased sensitivities of 85.8%, 77.0%, and 87.8%, respectively. When only samples tested positive by both ELISA were considered, the sensitivity decreased to 69.0%, 34.5%, and 34.8%, respectively. The simultaneous serological testing for antibodies recognizing M-, N-, and RBD-proteins may improve early detection of infected patients, although the RBD is sufficient to confirm previous infections and to monitor antibody titers after vaccination.

## Figures and Tables

**Table 1:** Performance characteristics of SARS-CoV-2 RBD- and M-protein based ELISA when probing for IgG or IgA antibodies recognizing either protein.

ELISA		RBD	M
IgG	Sensitivity		
	[+] <7 d	50.0% (9/18)	33.3% (6/18)
	[+] 7–14 d	75.0% (9/12)	41.7% (5/12)
	[+] >14 d	90.6% (77/85)	41.2% (35/85)
	Specificity		
	SARS-CoV-2 [−]	97.9% (92/94)	88.5% (46/52)
IgA	Sensitivity		
	[+] <7 d	83.3% (15/18)	50.0% (9/18)
	[+] 7–14 d	100.0% (12/12)	66.7% (7/12)
	[+] >14 d	98.8% (82/83)	31.8% (27/85)
	Specificity		
	SARS-CoV-2 [−]	84.0% (79/94)	88.5% (46/52)

## Data Availability

All data generated or analyzed during this study are included in this published article and its supplementary information files.

## Supplementary Materials

The following supporting information can be downloaded at: https://www.mdpi.com/article/10.3390/pathogens11121515/s1, Table S1: clinical parameters of patient serum samples; Table S2: clinical parameters of vaccinated and infected serum samples; Table S3: list of tested peptides with corresponding sequences; Figure S1: optimization of the S1 *E. coli* ELISA; Figure S2: optimization of the RBD *E. coli* ELISA; Figure S3: testing of DTT as additive for RBD and S1 *E. coli* ELISA; Figure S4: optimization of the M-protein *E. coli* ELISA; Figure S5: SDS-PAGE and corresponding immunoblots of purified SARS-CoV-2 proteins; Figure S6: calculated ROC-curves of IgG and IgA ELISAs; Figure S7: comparison between RBD, HEK monomer, dimer, and commercial RBDs; Figure S8: N-protein IgG ELISA of vaccinated and infected samples; Figure S9: RBD and S1 peptide IgG ELISA; Figure S10: comparison of IgA and IgG M-protein ELISA; Figure S11: M peptide IgG ELISA; Figure S12: advanced M IgG ELISA of the previously most promising peptides; Figure S13: scatter plot of the time- grouped samples with the best M-peptides; Figure S14: RBD- and N-protein ELISA of false positive samples; Figure S15: RBD- and S1-protein Glycostain; Figure S16: N-protein ELISA.
