# Supplementary material for: Evaluation of S- and M-Proteins Expressed in Escherichia coli and HEK Cells for Serological Detection of Antibodies in Response to SARS-CoV-2 Infections and mRNA-Based Vaccinations
Source: Pathogens. 2022 Dec 10;11(12):1515. doi: 10.3390/pathogens11121515 (PMC9782079; doi:10.3390/pathogens11121515)
Supplement: Supplementary file 1 [file pathogens-11-01515-s001.zip › pathogens-2044908-supplementary.pdf]

Supplementary Material

Evaluation of S- and M-Proteins Expressed in *Escherichia coli* and HEK Cells for Serological Detection of Antibodies in Re-Sponse to SARS-CoV-2 Infections and mRNA-Based Vaccinations

Mandy Schwarze [1,2,†], Ji Luo [1,2,3,†], Alexandra Brakel [1,2], Andor Krizsan [1,2], Nicole Lakowa [4], Thomas Grünewald [4], Claudia Lehmann [5], Johannes Wolf [6,7], Stephan Borte [6,7], Sanja Milkovska-Stamenova [1,2,3], Jörg Gabert [3], Markus Scholz [8,9], and Ralf Hoffmann [1,2,*]

[1] Institute of Bioanalytical Chemistry, Faculty of Chemistry and Mineralogy, Universität Leipzig, 04103 Leipzig, Germany
[2] Center for Biotechnology and Biomedicine, Universität Leipzig, 04103 Leipzig, Germany
[3] Adversis Pharma GmbH, 04103 Leipzig, Germany
[4] Klinik für Infektions- und Tropenmedizin, Klinikum Chemnitz gGmbH, 09113 Chemnitz, Germany
[5] Laboratory for Transplantation Immunology, Institute for Transfusion Medicine, University Hospital Leipzig, 04103 Leipzig, Germany
[6] Department of Laboratory Medicine, Hospital St. Georg gGmbH, 04129 Leipzig, Germany
[7] Immuno Deficiency Center Leipzig, Jeffrey Modell Diagnostic and Research Center for Primary Immunodeficiency Diseases, Hospital St. Georg gGmbH, 04129 Leipzig, Germany
[8] Institute for Medical Informatics, Statistics and Epidemiology, Universität Leipzig, 04107 Leipzig, Germany
[9] LIFE Research Center of Civilization Diseases, Universität Leipzig, 04103 Leipzig, Germany
* Correspondence: bioanaly@rz.uni-leipzig.de
† These authors contributed equally to this work.

Table of Contents

Optimization of ELISA S3

Table S1: Clinical parameters of patient serum samples S4

Table S2: Clinical parameters of vaccinated and infected serum samples S11

Table S3: List of tested peptides with corresponding sequences S14

Figure S1: Optimization of the S1 *E. coli* ELISA S15

Figure S2: Optimization of the RBD *E. coli* ELISA S16

Figure S3: Testing of DTT as additive for RBD and S1 *E. coli* ELISA S17

Figure S4: Optimization of the M-protein *E. coli* ELISA S18

Figure S5: SDS-PAGE and corresponding immunoblots of purified SARS-CoV-2 proteins S19

Figure S6: Calculated ROC-curves of IgG and IgA ELISAs S20

Figure S7: Comparison between RBD, HEK monomer, dimer, and commercial RBDs S21

Figure S8: N-protein IgG ELISA of vaccinated and infected samples S22

Figure S9: RBD and S1 peptide IgG ELISA S23

Figure S10: Comparison of IgA and IgG M-protein ELISA S24

Figure S11: M peptide IgG ELISA S25

Figure S12: Advanced M IgG ELISA of the previously most promising peptides S26

Figure S13: Scatter Plot of the time- grouped samples with the best M-peptides S27

Figure S14: RBD- and N-protein ELISA of false positive samples S28

Figure S15: RBD- and S1-protein Glycostain S29

Figure S16: N-protein ELISA S30

Optimization of ELISA

The indirect ELISA for anti-SARS-CoV-2 IgG antibodies was initially established for S1-protein expressed in *E. coli* to accelerate the assay development. First, the coating of the S1-protein was evaluated for carbonate buffer (pH 9.6), PBS (pH 7.4), and acetate buffer (pH 5.0) using an anti-His antibody and a negative pool (off-the-clot, sterile filtered; PAN-Biotech GmbH, Aidenbach, Germany) (Figure S1a). PBS provided the best ratio between anti-His antibody and negative pool. When S1-protein was coated in PBS buffer using a twofold dilution series from 1 µg/well to 1 ng/well, the OD_{450}-values decreased from 3.413 to 0.215 (Figure S1b) with the upper plateau of the curve starting at 63.0 ng/well. As lower quantities might increase the risk of pipetting errors during the dilution step, all further experiments used a coating of 250 ng/well S1-protein in PBS. When the same tests were repeated with the RBD-protein, again PBS appeared to provide the best coating conditions (Figure S2). When these conditions were tested on medium binding plates from Greiner Bio-One (Frickenhausen, Germany) or Brand GmbH + Co. Kg (Wertheim, Germany) and Maxisorp (Nunc, Roskilde, Denmark), the medium binding plate from Greiner provided the best performance based on the ratio of anti-His antibody and negative serum pool (Supplement, Figure S1c). These conditions optimized for recombinant S1- and RBD-proteins expressed in *E. coli* were also applied to S1- and RBD-proteins expressed in HEK cells.

To increase the selectivity, we additionally tested the *E. coli* proteins under reducing conditions in PBS supplemented with NaCl (200 mmol/L), urea (8 mol/L) and DTT (5 mmol/L) (Figure S3 and S4). However, no differences were observed for RBD-, S1-, and M-protein expressed in E. *coli*. Since the M-protein ELISA did not provide a good difference between positive and negative samples, the M-protein was coated in PBS and PBS supplemented with urea (4 mol/L) using the anti-His (10,000-fold dilution in Stabilzyme), a SARS-CoV-2 positive pool, and a negative pool. The best ratio of positive and negative pools was obtained when coating the M-protein (125 ng/well) in PBS supplemented with urea (4 mol/L; Figure S4).

Table S1. Clinical parameters of all patient serum samples included in the current study. The sample written in bold was obtained from a patient infected with SARS-CoV-2 variant B1.1.7.

Name	Origin	Sex	Age	Days after symptom onset	Days after PCR	RBD IgG (209)	M IgG (167)	M IgA (167)	RBD IgA (207)	N IgG (207)
C1	Chemnitz 2020	-	-		54	x	x	x	x	x
C2	Chemnitz 2020	-	-		50	x	x	x	x	x
C3	Chemnitz 2020	-	-		71	x	x	x	x	x
C4	Chemnitz 2020	-	-		50	x	x	x	x	x
C5	Chemnitz 2020	-	-		69	x	x	x	x	x
C6	Chemnitz 2020	-	-		70	x	x	x	x	x
C7	Chemnitz 2020	-	-		60	x	x	x	x	x
C8	Chemnitz 2020	-	-		79	x	x	x	x	x
C9	Chemnitz 2020	-	-		59	x	x	x	x	x
C10	Chemnitz 2020	-	-		67	x	x	x	x	x
C11	Chemnitz 2020	-	-		28	x	x	x	x	x
C12	Chemnitz 2020	-	-		68	x	x	x	x	x
C13.1	Chemnitz 2020	w	67		0	x	x	x	x	x
C13.2	Chemnitz 2020	w	67		10	x	x	x	x	x
C13.3	Chemnitz 2020	w	67		33	x	x	x	x	x
C14.1	Chemnitz 2020	m	68		1	x	x	x	x	x
C14.2	Chemnitz 2020	m	68		23	x	x	x	x	x
C15	Chemnitz 2020	m	80		36	x	x	x	x	x
C16	Chemnitz 2020	m	57		41	x	x	x	x	x
C17	Chemnitz 2020	w	51		24	x	x	x	x	x
C18	Chemnitz 2020	m	67		25	x	x	x	x	x
C19	Chemnitz 2020	w	64		25	x	x	x	x	x

C20	Chemnitz 2020	w	58	32	x	x	x	x	x
C21	Chemnitz 2020	w	62	22	x	x	x	x	x
C22	Chemnitz 2020	w	33	33	x	x	x	x	x
C23	Chemnitz 2020	w	62	6	x	x	x	x	x
C24	Chemnitz 2020	w	54	35	x	x	x	x	x
C25	Chemnitz 2020	m	56	32	x	x	x	x	x
C26	Chemnitz 2020	m	78	0	x	x	x	x	x
C27	Chemnitz 2020	w	88	5	x	x	x	x	x
C28	Chemnitz 2020	w	93	0	x	x	x	x	x
C29	Chemnitz 2020	m	66	27	x	x	x	x	x
C30	Chemnitz 2020	m	76	2	x	x	x	x	x
C31	Chemnitz 2020	m	82	5	x	x	x	x	x
C32.1	Chemnitz 2020	w	79	1	x	x	x	x	x
C32.2	Chemnitz 2020	w	79	20	x	x	x	x	x
C33	Chemnitz 2020	-	-	1	x	x	x	x	x
C34	Chemnitz 2020	m	66	9	x	x	x	x	x
C35	Chemnitz 2020	m	80	3	x	x	x	x	x
C36	Chemnitz 2020	m	81	16	x	x	x	x	x
C37.1	Chemnitz 2020	m	37	2	x	x	x	x	x
C37.2	Chemnitz 2020	m	37	26	x	x	x		x
C38.1	Chemnitz 2020	w	75	1	x	x	x	x	x
C38.2	Chemnitz 2020	w	75	15	x	x	x	x	x
C39	Chemnitz 2020	w	71	20	x	x	x	x	x
C40	Chemnitz 2020	m	75	4	x	x	x	x	x
C41	Chemnitz 2020	-	-	0	x	x	x	x	x
C42	Chemnitz 2020	-	-	19	x	x	x	x	x

SG1	St. Georg 2020	m	35	38	x	x	x	x	x
SG2	St. Georg 2020	f	54	43	x	x	x	x	x
SG3	St. Georg 2020	f	64	37	x	x	x	x	x
SG4	St. Georg 2020	m	25	3	x	x	x	x	x
SG5	St. Georg 2020	f	38	54	x	x	x	x	x
SG6	St. Georg 2020	m	78	40	x	x	x	x	x
SG7	St. Georg 2020	f	90	34	x	x	x	x	x
SG8	St. Georg 2020	m	47	44	x	x	x	x	x
SG9	St. Georg 2020	f	58	35	x	x	x	x	x
SG10	St. Georg 2020	m	55	36	x	x	x	x	x
SG11	St. Georg 2020	m	59	38	x	x	x	x	x
SG12	St. Georg 2020	m	54	44	x	x	x	x	x
SG13	St. Georg 2020	f	51	31	x	x	x	x	x
SG14	St. Georg 2020	m	53	49	x	x	x	x	x
SG15	St. Georg 2020	f	65	33	x	x	x	x	x
SG16	St. Georg 2020	m	24	23	x	x	x	x	x
SG17	St. Georg 2020	m	28	44	x	x	x	x	x
SG18	St. Georg 2020	f	64	53	x	x	x	x	x
SG19	St. Georg 2020	f	39	38	x	x	x	x	x
SG20	St. Georg 2020	m	63	51	x	x	x	x	x
SG21	St. Georg 2020	m	42	55	x	x	x	x	x
SG22	St. Georg 2020	f	52	35	x	x	x	x	x
SG23	St. Georg 2020	m	63	45	x	x	x	x	x
SG24	St. Georg 2020	m	17	45	x	x	x	x	x
SG25	St. Georg 2020	m	52	55	x	x	x	x	x
SG26	St. Georg 2020	f	54	47	x	x	x	x	x

SG27	St. Georg 2020	f	39	38	x	x	x	x	x
SG28	St. Georg 2020	m	15	38	x	x	x	x	x
SG29	St. Georg 2020	m	49	34	x	x	x	x	x
SG30	St. Georg 2020	m	50	48	x	x	x	x	x
SG31	St. Georg 2020	f	39	34	x	x	x	x	x
SG32	St. Georg 2020	f	34	35	x	x	x	x	x
SG33	St. Georg 2020	f	54	42	x	x	x	x	x
SG34	St. Georg 2020	m	42	34	x	x	x	x	x
SG35	St. Georg 2020	m	38	34	x	x	x	x	x
SG36	St. Georg 2020	f	40	34	x	x	x	x	x
SG37	St. Georg 2020	f	64	33	x	x	x	x	x
SG38	St. Georg 2020	m	53	31	x	x	x	x	x
SG39	St. Georg 2020	m	82	20	x	x	x	x	x
SG40	St. Georg 2020	f	84	11	x	x	x	x	x
SG41	St. Georg 2020	f	81	20	x	x	x	x	x
SG42	St. Georg 2020	m	82	27	x	x	x	x	x
SG43	St. Georg 2020	f	78	30	x	x	x	x	x
SG44	St. Georg 2020	m	88	22	x	x	x	x	x
SG45	St. Georg 2020	f	89	8	x	x	x	x	x
SG46	St. Georg 2020	f	64	12	x	x	x	x	x
SG47	St. Georg 2020	f	81	10	x	x	x	x	x
SG48	St. Georg 2020	f	81	1	x	x	x	x	x
SG49	St. Georg 2020	m	25	32	x	x	x	x	x
SG50	St. Georg 2020	m	54	30	x	x	x	x	x
SG51	St. Georg 2020	m	60	12	x	x	x	x	x
SG52	St. Georg 2020	m	68	15	x	x	x		

SG53	St. Georg 2020	f	47	21	x	x	x	x	x
SG54	St. Georg 2020	m	55	14	x	x	x	x	x
SG55	St. Georg 2021	f	77	52	x	x	x	x	x
**SG56**	**St. Georg 2021**	**m**	**75**	**8**	**x**	**x**	**x**	**x**	**x**
SG57	St. Georg 2020	f	64	8	x	x	x	x	x
SG58	St. Georg 2020	m	88	3	x	x	x	x	x
SG59	St. Georg 2020	m	83	14	x	x	x	x	
SG60	St. Georg 2020	m	31	29	x	x	x	x	x
SG61	St. Georg 2020	f	78	9	x	x	x	x	x
SG62	St. Georg 2020	f	34	35	x	x	x	x	x
SG63	St. Georg 2020	f	40	54	x	x	x	x	x
SG64	St. Georg 2020	f	37	33	x	x	x	x	x
SG65	St. Georg 2020	f	46	33	x	x	x	x	x
SG66	St. Georg 2020	m	43	67	x	x	x	x	x
SG67	St. Georg 2020	m	56	26	x	x	x	x	x
L1	LIFE 2015	f	75			x	x		
L2	LIFE 2015	m	57			x	x		
L3	LIFE 2015	f	65			x	x		
L4	LIFE 2015	m	76			x	x		
L5	LIFE 2015	f	75			x	x		
L6	LIFE 2015	f	53			x	x		
L7	LIFE 2015	m	51			x	x		
L8	LIFE 2015	m	77			x	x		
L9	LIFE 2015	m	74			x	x		
L10	LIFE 2015	m	58			x	x		
L11	LIFE 2015	m	63			x	x		
L12	LIFE 2015	m	64			x	x		
L13	LIFE 2015	m	50			x	x		
L14	LIFE 2015	m	67			x	x		
L15	LIFE 2015	m	62			x	x		
L16	LIFE 2015	f	56			x	x		
L17	LIFE 2015	m	76			x	x		
L18	LIFE 2015	m	73			x	x		
L19	LIFE 2015	f	59			x	x		
L20	LIFE 2015	m	79			x	x		

L21	LIFE 2015	m	70		x	x		
L22	LIFE 2015	m	78		x	x		
L23	LIFE 2015	m	77		x	x		
L24	LIFE 2015	m	71		x	x		
L25	LIFE 2015	f	73		x	x		
L26	LIFE 2015	m	56		x	x		
L27	LIFE 2015	m	76		x	x		
L28	LIFE 2015	m	51		x	x		
L29	LIFE 2015	m	59		x	x		
L30	LIFE 2015	f	60		x	x		
L31	LIFE 2015	m	58		x	x		
L32	LIFE 2015	m	52		x	x		
L33	LIFE 2015	f	57		x	x		
L34	LIFE 2015	m	60		x	x		
L35	LIFE 2015	m	54		x	x		
L36	LIFE 2015	f	60		x	x		
L37	LIFE 2015	f	54		x	x		
L38	LIFE 2015	f	65		x	x		
L39	LIFE 2015	m	67		x	x		
L40	LIFE 2015	m	72		x	x		
L41	LIFE 2015	m	67		x	x		
L42	LIFE 2015	m	54		x	x		
L43	LIFE 2015	m	69		x	x		
L44	LIFE 2015	f	67		x	x		
L45	LIFE 2015	f	67		x	x		
L46	LIFE 2015	m	64		x	x		
L47	LIFE 2015	m	53		x	x		
L48	LIFE 2015	m	70		x	x		
L49	LIFE 2015	f	54		x	x		
L50	LIFE 2015	m	73		x	x		
L51	LIFE 2015	m	56		x	x		
L52	LIFE 2015	f	60		x	x		
L53	LIFE 2015	f	43	x			x	x
L54	LIFE 2015	m	50	x			x	x
L55	LIFE 2015	m	75	x			x	x
L56	LIFE 2015	m	57	x			x	x
L57	LIFE 2015	m	45	x			x	x
L58	LIFE 2015	f	46	x			x	x
L59	LIFE 2015	m	41	x			x	x
L60	LIFE 2015	f	44	x			x	x
L61	LIFE 2015	f	47	x			x	x
L62	LIFE 2015	f	42	x			x	x
L63	LIFE 2015	m	49	x			x	x
L64	LIFE 2015	f	45	x			x	x
L65	LIFE 2015	m	45	x			x	x
L66	LIFE 2015	f	48	x			x	x
L67	LIFE 2015	f	67	x			x	x

L68	LIFE 2015	f	62	x	x	x
L69	LIFE 2015	f	48	x	x	x
L70	LIFE 2015	f	41	x	x	x
L71	LIFE 2015	f	48	x	x	x
L72	LIFE 2015	f	51	x	x	x
L73	LIFE 2015	f	49	x	x	x
L74	LIFE 2015	m	50	x	x	x
L75	LIFE 2015	f	62	x	x	x
L76	LIFE 2015	f	63	x	x	x
L77	LIFE 2015	f	67	x	x	x
L78	LIFE 2015	f	60	x	x	x
L79	LIFE 2015	f	76	x	x	x
L80	LIFE 2015	m	48	x	x	x
L81	LIFE 2015	f	65	x	x	x
L82	LIFE 2015	f	41	x	x	x
L83	LIFE 2015	f	57	x	x	x
L84	LIFE 2015	m	68	x	x	x
L85	LIFE 2015	f	76	x	x	x
L86	LIFE 2015	m	65	x	x	x
L87	LIFE 2015	f	73	x	x	x
L88	LIFE 2015	m	66	x	x	x
L89	LIFE 2015	f	78	x	x	x
L90	LIFE 2015	m	78	x	x	x
L91	LIFE 2015	f	45	x	x	x
L92	LIFE 2015	f	72	x	x	x
L93	LIFE 2015	f	49	x	x	x
L94	LIFE 2015	f	58	x	x	x
L95	LIFE 2015	f	63	x	x	x
L96	LIFE 2015	m	45	x	x	x
L97	LIFE 2015	f	43	x	x	x
L98	LIFE 2015	m	59	x	x	x
L99	LIFE 2015	m	70	x	x	x
L100	LIFE 2015	f	55	x	x	x
L101	LIFE 2015	m	74	x	x	x
L102	LIFE 2015	m	57	x	x	x
L103	LIFE 2015	f	68	x	x	x
L104	LIFE 2015	f	67	x	x	x
L105	LIFE 2015	m	43	x	x	x
L106	LIFE 2015	m	51	x	x	x
L107	LIFE 2015	f	44	x	x	x
L108	LIFE 2015	f	74	x	x	x
L109	LIFE 2015	f	63	x	x	x
L110	LIFE 2015	f	77	x	x	x
L111	LIFE 2015	m	57	x	x	x
L112	LIFE 2015	m	57	x	x	x
L113	LIFE 2015	f	45	x	x	x
L114	LIFE 2015	m	52	x	x	x

L115	LIFE 2015	f	64			x			x	x
L116	LIFE 2015	f	51			x			x	x
L117	LIFE 2015	m	60			x			x	x
L118	LIFE 2015	f	75			x			x	x
L119	LIFE 2015	f	73			x			x	x
L120	LIFE 2015	m	52			x			x	x
L121	LIFE 2015	f	47			x			x	x
L122	LIFE 2015	m	58			x			x	x
L123	LIFE 2015	m	43			x			x	x
L124	LIFE 2015	f	66			x			x	x
L125	LIFE 2015	f	59			x			x	x
L126	LIFE 2015	f	65			x			x	x
L127	LIFE 2015	m	50			x			x	x
L128	LIFE 2015	m	49			x			x	x
L129	LIFE 2015	f	55			x			x	x
L130	LIFE 2015	f	51			x			x	x
L131	LIFE 2015	m	59			x			x	x
L132	LIFE 2015	m	50			x			x	x
L133	LIFE 2015	m	41			x			x	x
L134	LIFE 2015	m	60			x			x	x
L135	LIFE 2015	f	52			x			x	x
L136	LIFE 2015	m	45			x			x	x
L137	LIFE 2015	m	66			x			x	x
L138	LIFE 2015	f	51			x			x	x
L139	LIFE 2015	f	30			x			x	x
L140	LIFE 2015	f	42			x			x	x
L141	LIFE 2015	m	65			x			x	x
L142	LIFE 2015	m	56			x			x	x
L143	LIFE 2015	f	55			x			x	x
L144	LIFE 2015	f	50			x			x	x
L145	LIFE 2015	f	59			x			x	x
L146	LIFE 2015	f	51			x			x	x

Table S2: Clinical parameters of serum samples collected from vaccinated persons.

Name	Sex	Age	Days after 1st vacc.	Days after 2nd vacc.	Days after 3rd vacc.	Days after PCR	1st and 2nd vaccine	3rd vaccine
b1.0	f	27	-	-	-	-	BNT162b2	BNT162b2
b1.1	f	27	17	-	-	-	BNT162b2	BNT162b2
b1.3	f	27	223	202	-	-	BNT162b2	BNT162b2
b1.4	f	27	241	220	15	-	BNT162b2	BNT162b2
b1.5	f	27	311	290	85	-	BNT162b2	BNT162b2
b1.i	f	27	361	340	135	34	BNT162b2	BNT162b2
b2.0	m	36	-	-	-	-	BNT162b2	BNT162b2
b2.2	m	36	27	-	-	-	BNT162b2	BNT162b2
b2.3	m	36	172	151	-	-	BNT162b2	BNT162b2
b2.5	m	36	318	297	68	-	BNT162b2	BNT162b2
b3.0	m	29	-	-	-	-	BNT162b2	BNT162b2
b3.1	m	29	15	-	-	-	BNT162b2	BNT162b2
b3.3	m	29	171	27	-	-	BNT162b2	BNT162b2
b3.5	m	29	317	173	70	-	BNT162b2	BNT162b2
b4.1	f	30	17	-	-	-	BNT162b2	BNT162b2
b4.2	f	30	26	5	-	-	BNT162b2	BNT162b2
b4.3	f	30	164	143	-	-	BNT162b2	BNT162b2
b4.4	f	30	253	232	30	-	BNT162b2	BNT162b2
b4.5	f	30	317	296	94	-	BNT162b2	BNT162b2
b5.1	f	28	14	-	-	-	BNT162b2	BNT162b2
b5.3	f	28	170	141	-	-	BNT162b2	BNT162b2
b5.5	f	28	316	287	84	-	BNT162b2	BNT162b2
b6.2	f	59	28	7	-	-	BNT162b2	BNT162b2
b6.3	f	59	171	150	-	-	BNT162b2	BNT162b2
b6.4	f	59	229	208	16	-	BNT162b2	BNT162b2
b6.5	f	59	317	296	104	-	BNT162b2	BNT162b2
b7.1	m	58	15	-	-	-	BNT162b2	BNT162b2
b7.2	m	58	29	8	-	-	BNT162b2	BNT162b2

b7.3	m	58	174	153	-	-	BNT162b2	BNT162b2
b7.4	m	58	220	199	14	-	BNT162b2	BNT162b2
b7.5	m	58	322	301	116	-	BNT162b2	BNT162b2
b7.i	m	58	373	352	167	15	BNT162b2	BNT162b2
b8.5	f	45	314	293	102	-	BNT162b2	BNT162b2
b9.5	f	45	304	283	104	-	BNT162b2	BNT162b2
b10.5	m	26	244	216	63	-	BNT162b2	BNT162b2
b11.5	f	26	285	264	102	-	BNT162b2	BNT162b2
b12.5	f	50	305	284	96	-	BNT162b2	BNT162b2
b13.5	m	33	307	283	102	-	BNT162b2	BNT162b2
b14.5	m	41	315	294	102	-	BNT162b2	BNT162b2
m1.0	m	36	-	-	-	-	mRNA-1273	mRNA-1273
m1.1	m	36	7	-	-	-	mRNA-1273	mRNA-1273
m1.5	m	36	295	267	91	-	mRNA-1273	mRNA-1273
m2.0	m	28	2	-	-	-	mRNA-1273	
m2.1	m	28	16	-	-	-	mRNA-1273	
m2.2	m	28	37	9	-	-	mRNA-1273	
m3.0	m	33	-	-	-	-	mRNA-1273	mRNA-1273
m3.1	m	33	12	-	-	-	mRNA-1273	mRNA-1273
m3.2	m	33	33	5	-	-	mRNA-1273	mRNA-1273
m3.3	m	33	212	184	-	-	mRNA-1273	mRNA-1273
m3.4	m	33	230	202	18	-	mRNA-1273	mRNA-1273
m3.5	m	33	300	272	88	-	mRNA-1273	mRNA-1273
m3.i	m	33	350	322	138	49	mRNA-1273	mRNA-1273
m4.0	f	28	-	-	-	-	mRNA-1273	
m4.1	f	28	14	-	-	-	mRNA-1273	
m4.2	f	28	35	7	-	-	mRNA-1273	
m5.5	f	60	316	288	119	-	mRNA-1273	mRNA-1273
m6.2	m	31	45	17	-	-	mRNA-1273	
m7.0	f	27	2	-	-	-	mRNA-1273	
m7.1	f	27	16	-	-	-	mRNA-1273	

m7.2	f	27	37	9	-	-	mRNA-1273	
mb1.0	m	32	-	-	-	-	mRNA-1273	BNT162b2
mb1.1	m	32	14	-	-	-	mRNA-1273	BNT162b2
mb1.2	m	32	35	7	-	-	mRNA-1273	BNT162b2
mb1.3	m	32	154	126	-	-	mRNA-1273	BNT162b2
mb1.4	m	32	214	186	15	-	mRNA-1273	BNT162b2
mb1.5	m	32	302	274	103	-	mRNA-1273	BNT162b2
mb2.0	f	25	-	-	-	-	mRNA-1273	BNT162b2
mb2.1	f	25	12	-	-	-	mRNA-1273	BNT162b2
mb2.2	f	25	33	5	-	-	mRNA-1273	BNT162b2
mb2.3	f	25	212	184	-	-	mRNA-1273	BNT162b2
mb2.4	f	25	230	202	18	-	mRNA-1273	BNT162b2
mb2.5	f	25	300	272	88	-	mRNA-1273	BNT162b2
mb2.i	f	25	350	322	138	49	mRNA-1273	BNT162b2
mb3.1	m	22	14	-	-	-	mRNA-1273	BNT162b2
mb3.2	m	22	35	7	-	-	mRNA-1273	BNT162b2
mb3.3	m	22	168	140	-	-	mRNA-1273	BNT162b2
mb3.4	m	22	224	196	13	-	mRNA-1273	BNT162b2
mb3.5	m	22	312	284	101	-	mRNA-1273	BNT162b2
mb4.1	m	24	14	-	-	-	mRNA-1273	BNT162b2
mb4.2	m	24	35	7	-	-	mRNA-1273	BNT162b2
mb4.4	m	24	224	196	13	-	mRNA-1273	BNT162b2
mb4.5	m	24	312	284	101	-	mRNA-1273	BNT162b2
mb4.i	m	24	363	335	152	24	mRNA-1273	BNT162b2
mb5.5	m	32	308	280	102	-	mRNA-1273	BNT162b2

Table S3. List of tested peptides with corresponding sequences, positions in the S1- and M-protein, and peptide purity. Peptides were synthesized as C-terminal acids or amides (denoted as NH_2).

Peptide-ID	Sequence	Position	Purity
S1	MFVFLVLLPLVSSQCVNLT	S1-19	98%
S22	TQLPPAYTNSFTRG-NH_2	S22-35	>95%
S182	KQGNFKNLREFVFK-NH_2	S182-195	>95%
S352	AWNRKRISNCVAD-NH_2	S352-364	94%
S452	LYRLFRKSNLKPFE-NH_2	S452-465	>95%
S655	VNNSYECDIPIGAGICA-NH_2	S655-672	95%
S782	AQVKQIYKTPPIKDFG-NH_2	S782-798	98%
S803	SQILPDPSKPSKRSFIEDL-NH_2	S803-821	>95%
S811	KPSKRSFIEDLL-NH_2	S811-822	98%
S881	TITSGWTFGAGAAL-NH_2	S881-894	>95%
S1148	HLMSFPQSAPH-NH_2	S1148-1158	99%
S1191	KNLNESLIDLQELG-NH_2	S1191-1204	>95%
RBD392	FTNVYADSFVIRGDEVRQIAPGQTGKIADY-NH_2	S392-421	92%
RBD481	NGVEGFNCYFPLQSYGFQPTNGVGYQPYRV-NH_2	S481-510	98%
M1	MADSNGTITVEELKKLLE	M1-18	98%
M4	SNGTITVEELKKLLEQWN	M4-21	>95%
M101	RLFARTRSMWSFNP	M101-114	83%
M173	SRTLSYYKLGASQR	M173-186	>95%
M188	AGDSGFAAYSRYR	M188-200	94%
M204	YKLNTDHSSSSDNIALLVQ	M204-222	98%

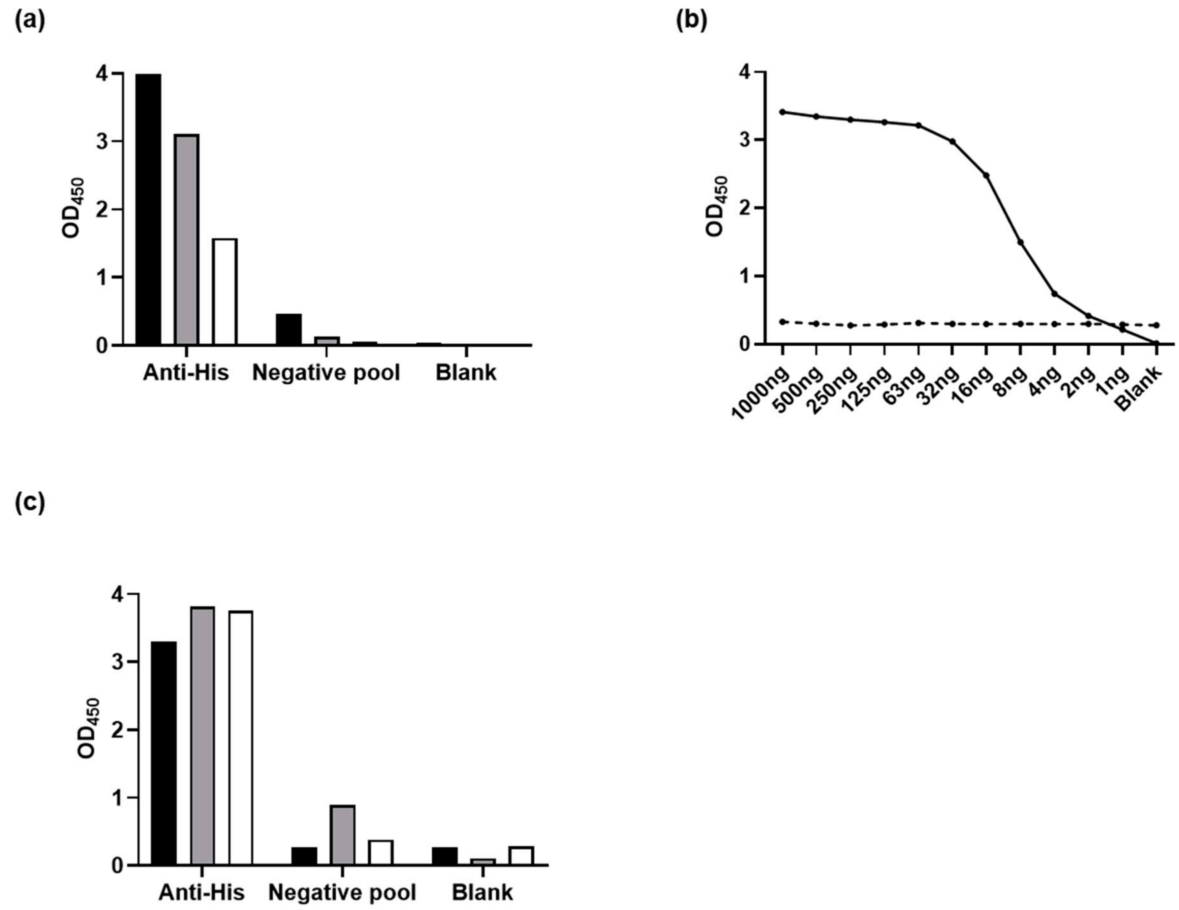

Figure S1. Optimization of the S1-protein (expressed in *E. coli*) ELISA by testing different (a) coating buffers including carbonate buffer (Black, pH 9.6), PBS (grey, pH 7.4) and acetate buffer (white, pH 5.0); (b) S1-protein quantities with positive (solid line) and negative (dash line) pool; (c) plate types including medium binding from Greiner (Black), medium binding from Brand (Grey), and Maxisorp from Nunc (white) using anti-His (10,000-fold dilution in Stabilzyme) and negative pool (100-fold dilution in Assay diluent).

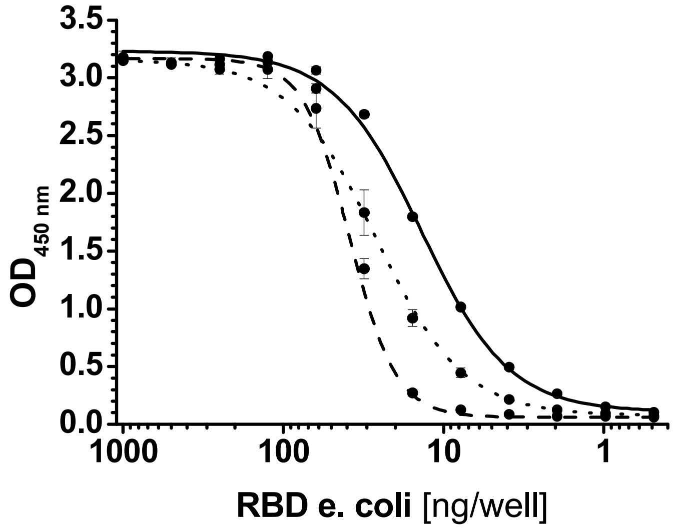

Figure S2. Optimization of the RBD *E. coli* ELISA by testing different coating buffers including PBS (line, pH 7.4), acetate (dashed, pH 5.0) and carbonate (pointed, pH 9.2) using anti-His antibody (10,000-fold dilution in Stabilzyme).

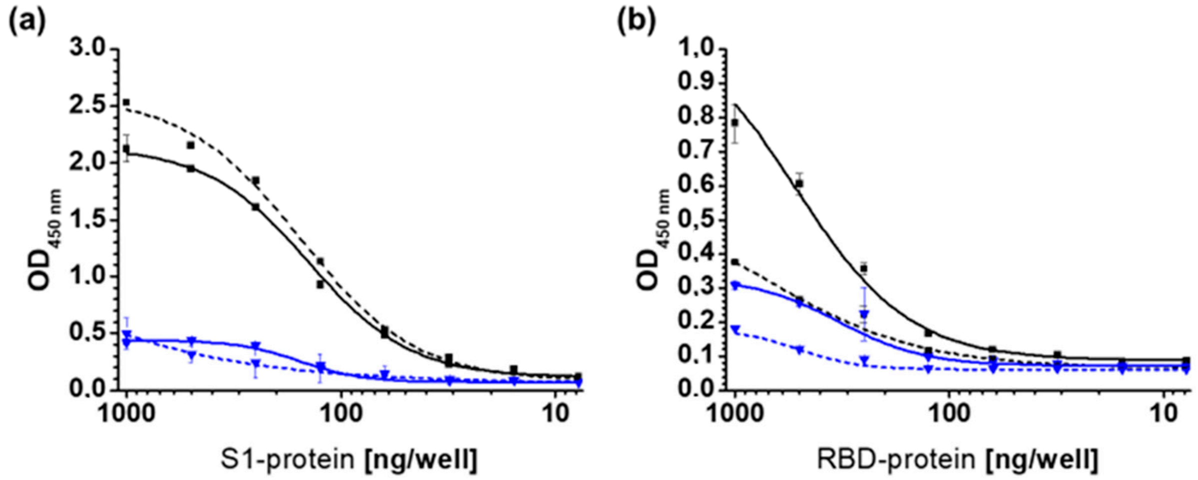

Figure S3. Absorbance recorded in ELISA using (a) S1- and (b) RBD-proteins expressed in *E. coli* and coating buffers without (solid line) and with DTT (dashed lines) using a positive (black) and negative (blue) serum pool as secondary antibody (25,000-fold dilution).

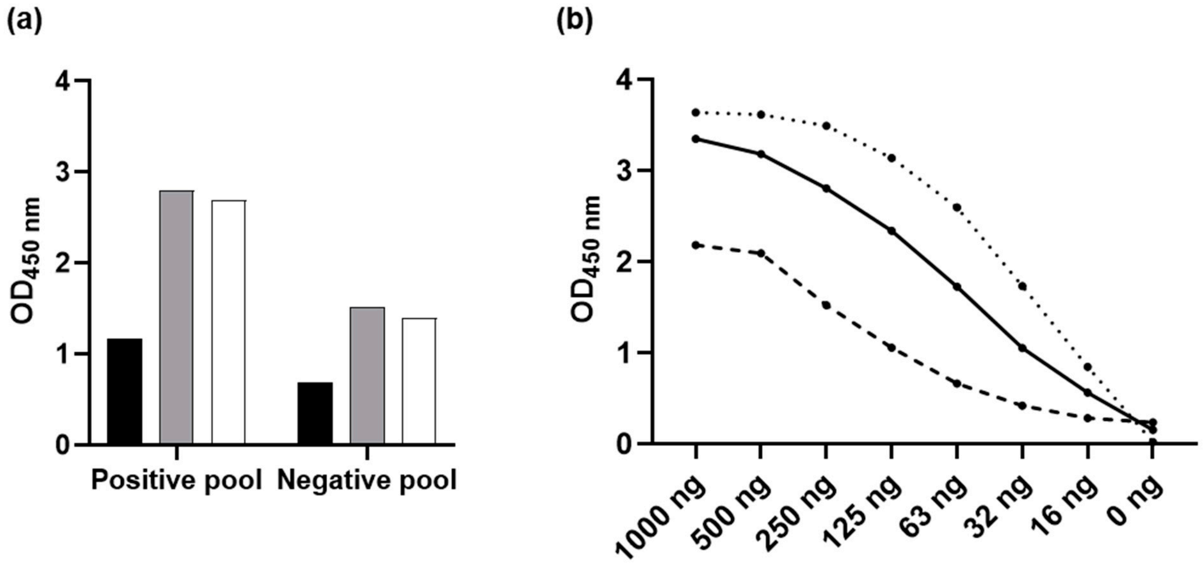

Figure S4. Absorbance recorded in ELISA when coating the full-length M-protein (1 µg/well) expressed in *E. coli* from (a) PBS (black bars), PBS supplemented with urea (4 mol/L; grey bars), and PBS supplemented with urea (4 mol/L) and DTT (5 mmol/L; white bars) or (b) in a twofold dilution series in PBS probed with an anti-His antibody (dotted line, 10,000-fold dilution in Stabilzyme), a positive pool (solid line, 100-fold dilution in Assay diluent), and a negative pool (dashed line, 100-fold dilution in Assay diluent).

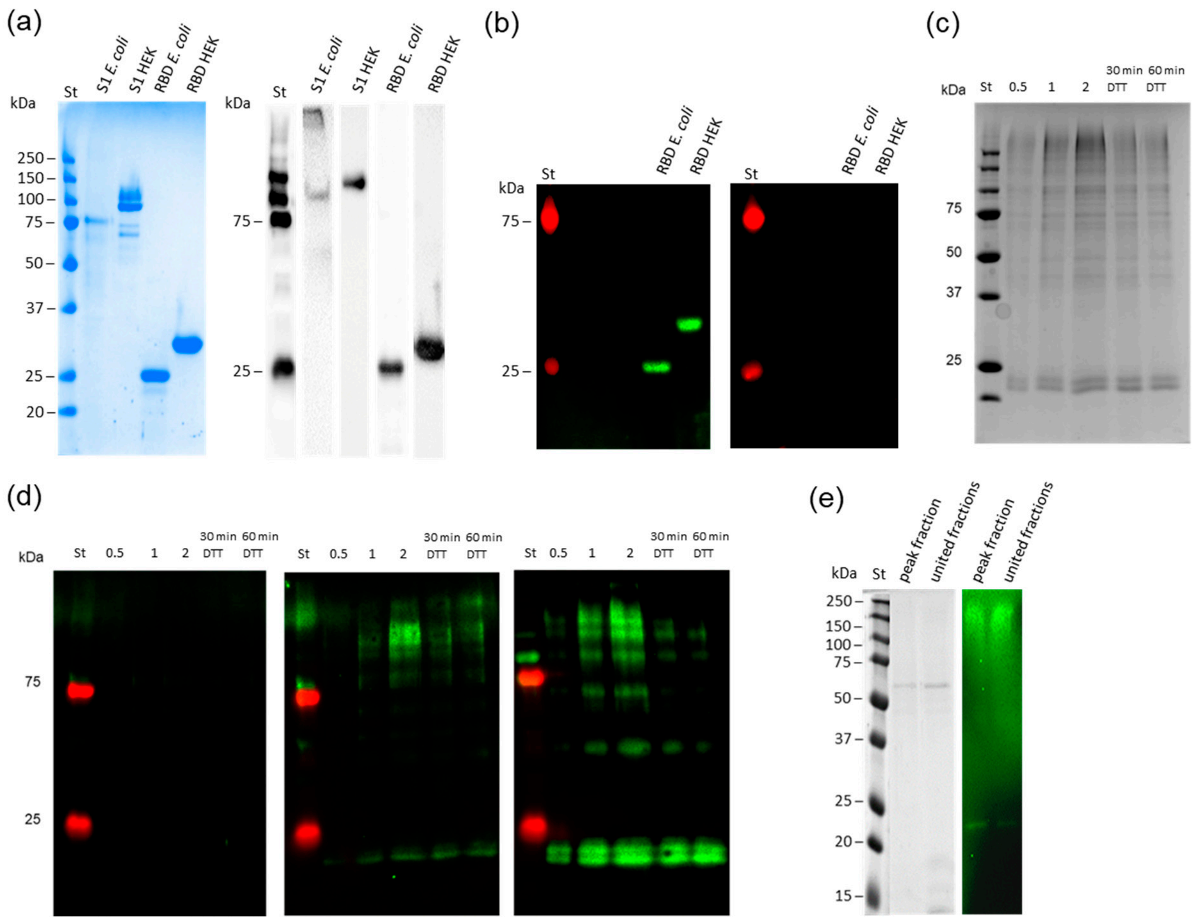

Figure S5. SDS-PAGE and corresponding immunoblots of purified SARS-CoV-2 S1-, RBD-, and M-proteins. A protein standard with the molecular weights indicated on the left side of the gels and immunoblots was loaded in the first lane. Gels were stained with Coomassie Brilliant Blue R254. S1- and RBD-proteins (1 µg each) expressed in *E. coli* or HEK cells (a, left) and the corresponding immunoblots probed with an anti-His-tag antibody (a, right) and with SARS-CoV-2 positive (b, left) or negative serum pools (b, right). SDS-PAGE of the *E. coli* M-proteins loaded at different quantities indicated on top of each lane or after reduction of M-protein (1 µg) using dithiothreitol (5 mmol/L, 50 °C; c) and the corresponding immunoblots probed with negative serum (d, left) or positive serum pools (d, middle) and an anti-His-tag antibody (d, right). The positive and negative pools were 10,000-fold and the anti-His-tag antibody 15,000-fold diluted in PBS. SDS-PAGE (e, left) and immunoblot (e, right) of the HEK M-protein loaded with the peak fraction and united fractions of the last purification step (size-exclusion chromatography). The C-terminal TwinStrep tagged M-protein was detected via Strep-HRP derivate.

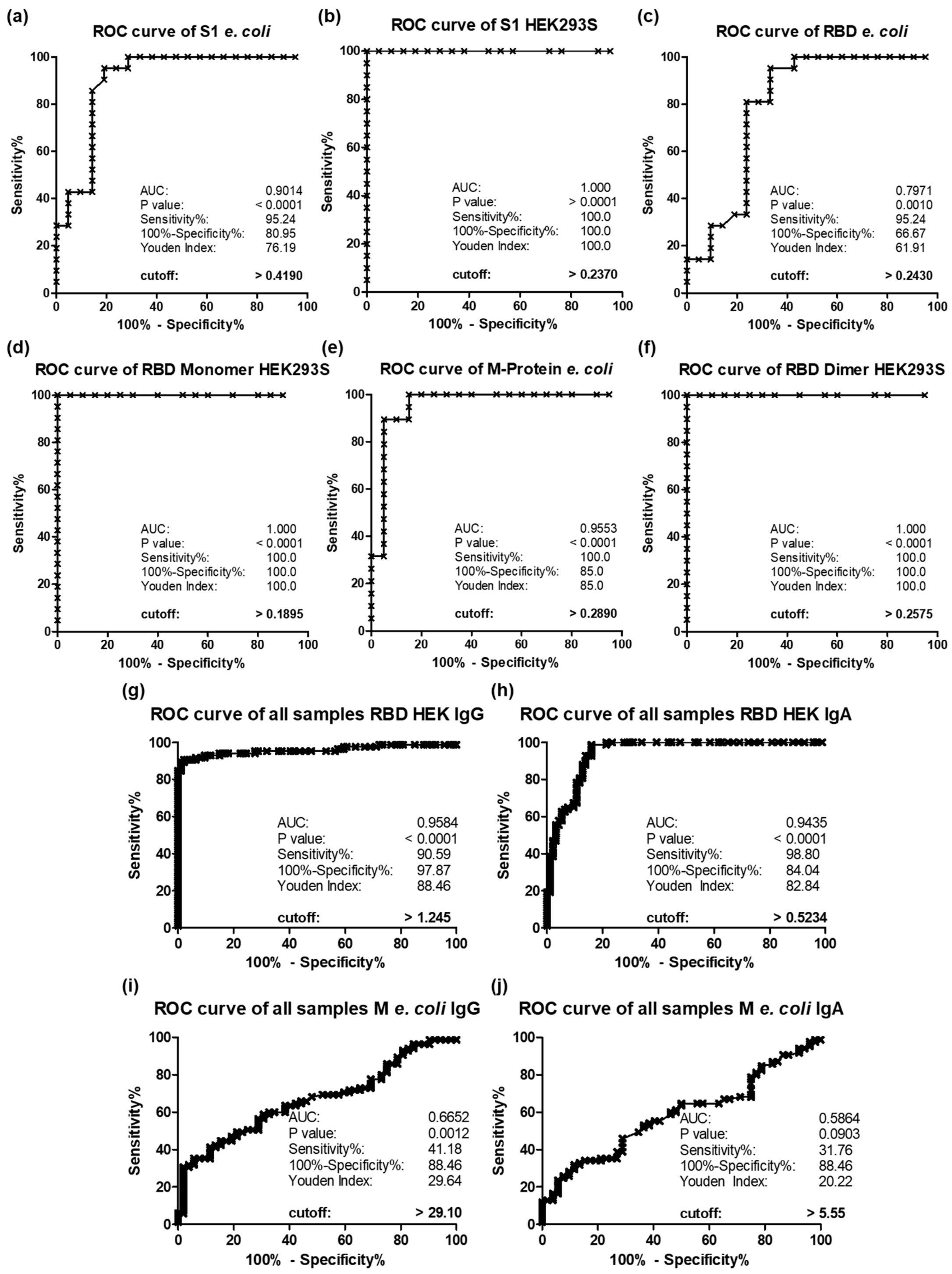

Figure S6. Calculated ROC-curves (GraphPad Prism 5.02) including cutoff, sensitivity and specificity for all established protein ELISA tested for a set of 20 positive and 20 control samples (a-f) and the whole serum sample set consisting of 115 and 94 samples (g), 113 and 94 samples (g), 115 and 52 samples (i) and (j), respectively. The ELISA used S1-proteins expressed in *E. coli* (a) or HEK293 cells (b), RBD-proteins expressed in *E. coli* (c) or HEK293S cells (d), M-protein expressed in *E. coli* (e), the RBD dimer expressed in HEK293S cells (f) and the whole sample set.

The ELISA probed the sera for anti-protein IgG antibodies (a-g, i) and IgA (h, j). Cutoffs were determined using the Youden index.

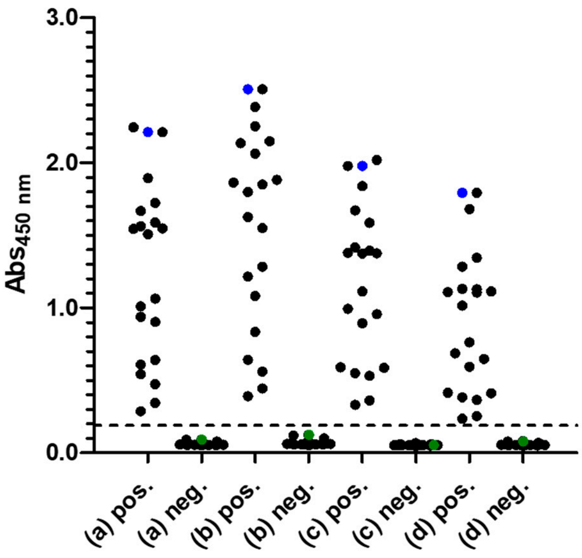

Figure S7. Distribution of OD_{450}-values obtained from 20 SARS-CoV-2 positive and 20 negative human serum samples (black dots) comparing monomeric (a) and dimeric RBD-protein versions expressed in HEK cells (b) and commercial RBD-proteins obtained from GenScript Biotech Corp (New Jersey, USA) (c) and Biotype GmbH (Dresden, Germany) (d) probed for IgG antibodies. Positive and negative pools are indicated as blue and green dots, respectively. The cutoff of the in-house ELISA using monomeric RBD is indicated as a dotted line (0.1895).

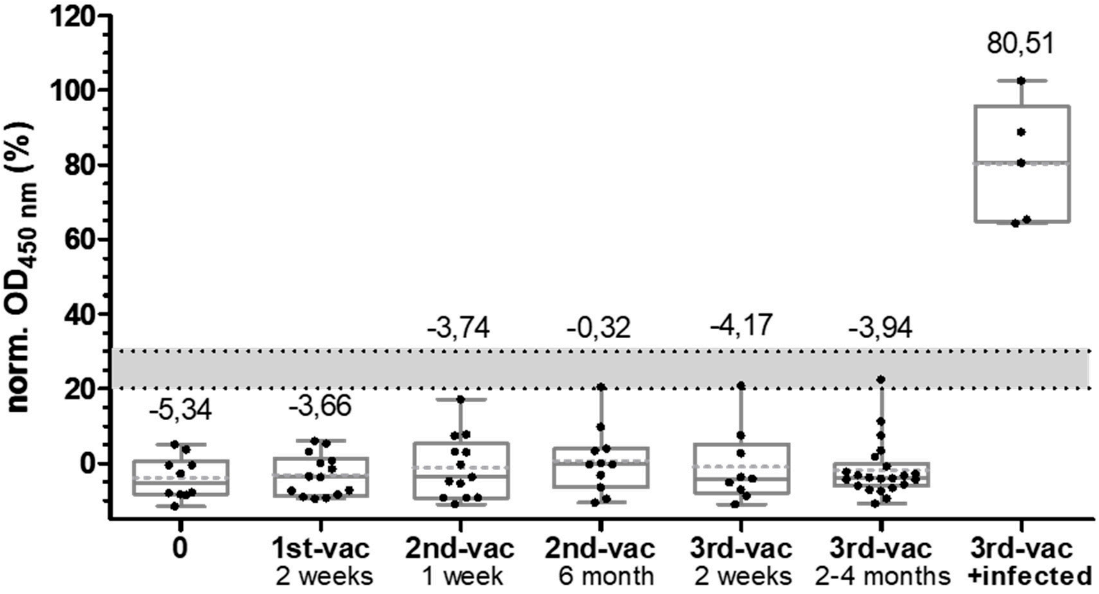

Figure S8. Distribution of normalized OD_{450}-values obtained from serum samples collected from persons vaccinated with mRNA vaccines BNT162b2 or mRNA-1273 and confirmed SARS-CoV-2 infections tested in ELISA for IgG antibodies recognizing N-protein expressed in *E. coli*. Positive (100%) and negative pools (0%) tested on each plate were used for normalization. The cutoff range is indicated as grey area between the dotted lines.

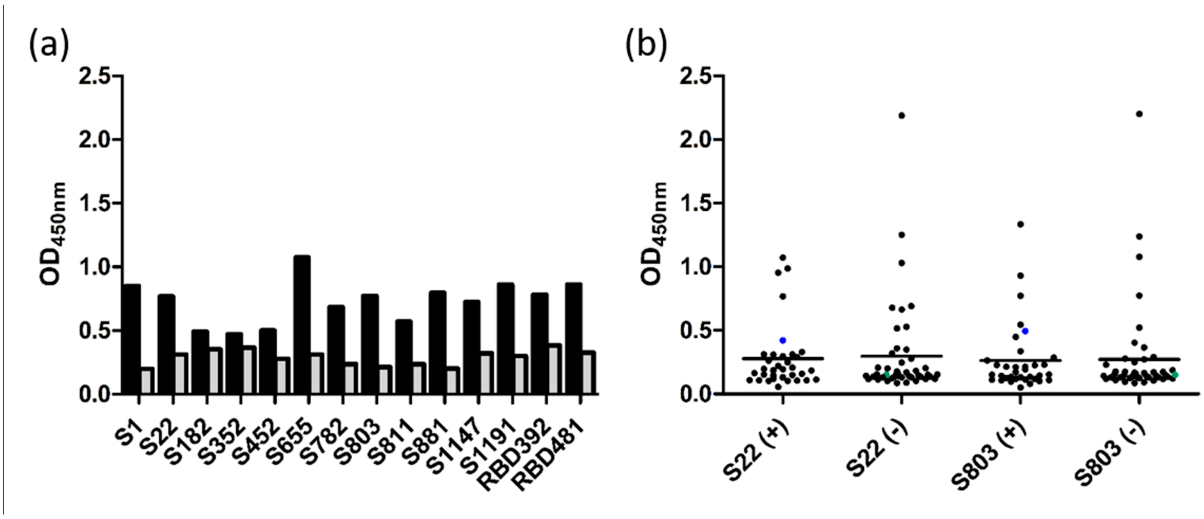

Figure S9: OD_{450}-values obtained for S- and RBD-peptides tested in ELISA for IgG antibodies using (a) pooled positive (black) and negative (grey) sera or (b) sera of infected (+) or non-infected (-) patients. The positive and negative pools are indicated as blue and green dots (b), respectively.

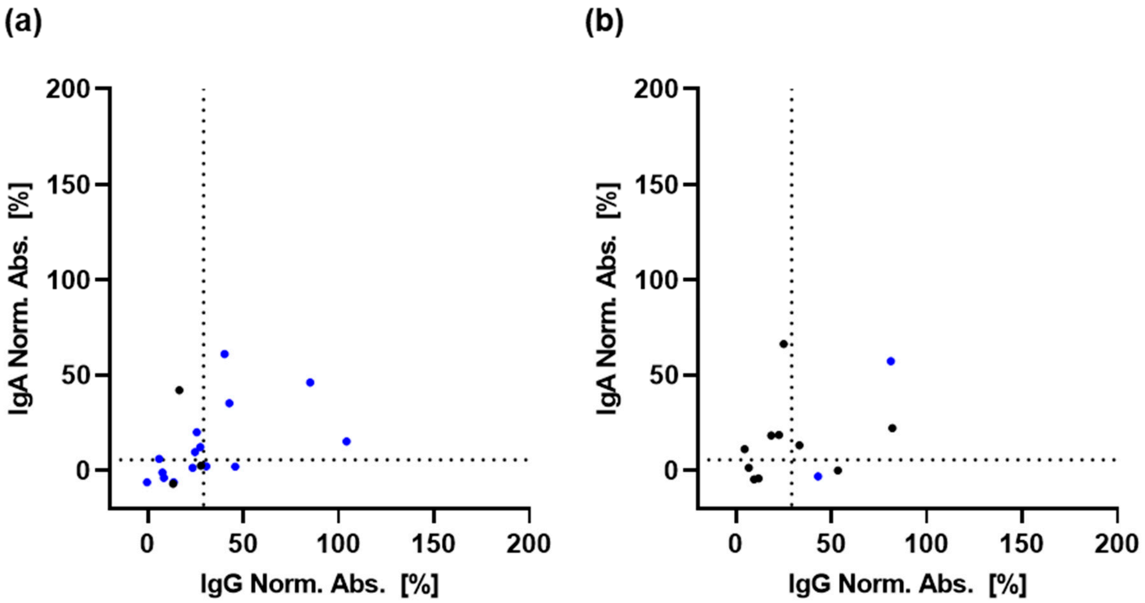

Figure S10. Comparison of normalized IgA and IgG absorbances obtained by the M-protein ELISA for patient samples of groups 1 (a) and 2 (b) based on symptom onset (black) or PCR confirmation (blue).

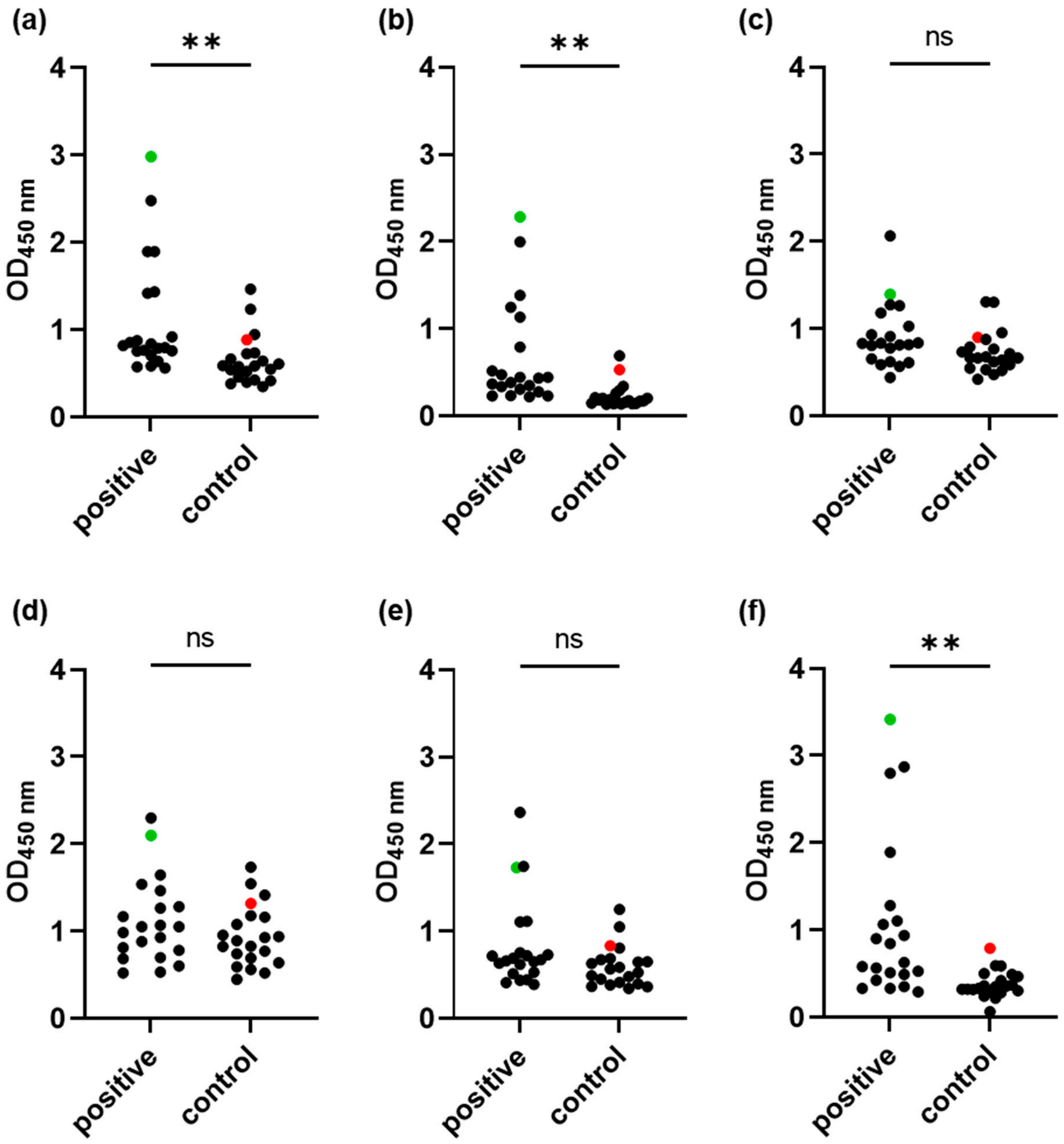

Figure S11. OD450-values obtained by testing 20 positive and 20 control serum samples in peptide ELISAs for IgG antibodies recognizing (a) M1, (b) M4, (c) M101, (d) M179, (e) M188, and (f) M204 peptides. The positive and negative pools are indicated as blue and green dots, respectively. Asterisk indicates significance ** P ≤ 0.01, ns: not significant.

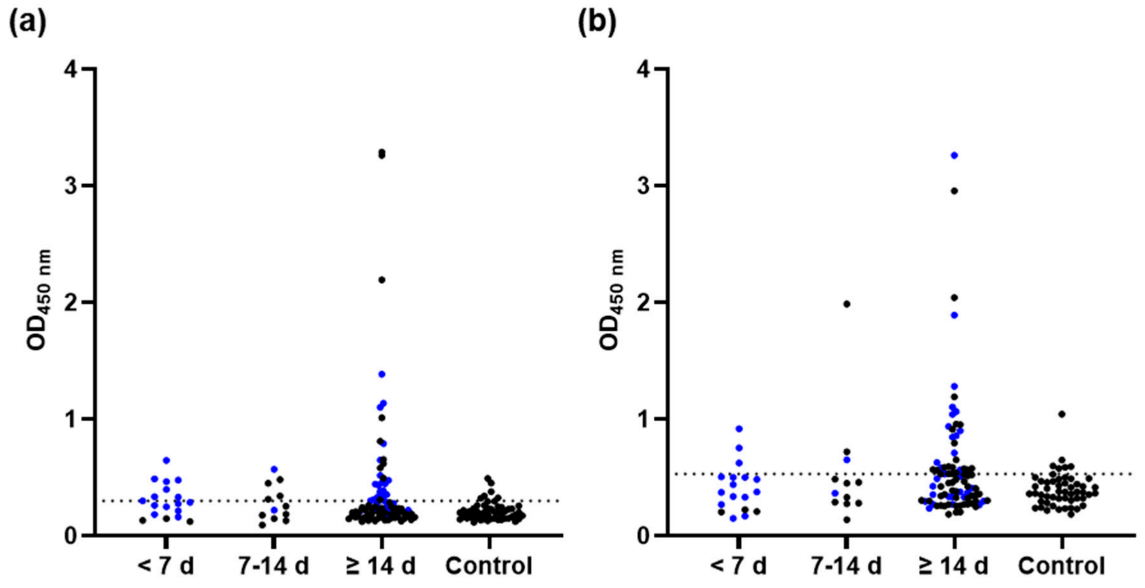

Figure S12. OD450-values obtained by testing (a) M4 and (b) M204 peptides corresponding to residues 4-21 and 204-222 of M-protein in the peptide-ELISA probed for IgG antibodies using all serum samples of groups 1 to 3 based on symptom onset (black dots, N = 48) or PCR test confirmation (blue dots, N = 67) and 52 control samples. The cutoff values of the M4 and M204 ELISA were 0.27 and 0.51, respectively, based on ROC curve analyses.

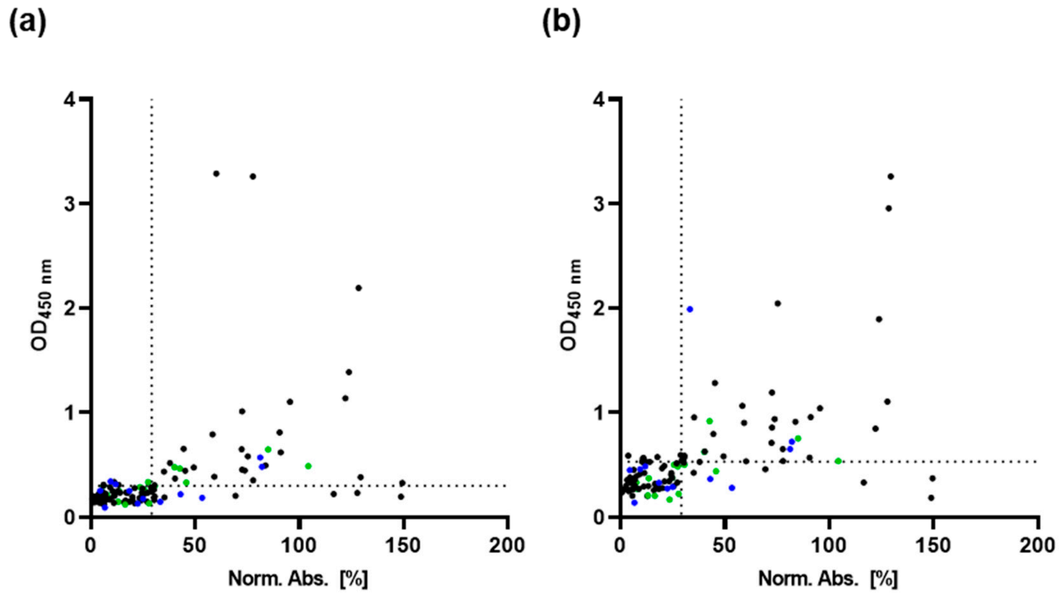

Figure S13. Scatter plots displaying the OD_{450}-values obtained for all serum samples of groups 1 (green dots), 2 (blue dots), and 3 (black dots) by using the ELISA results obtained for (a) M4 or (b) M204 peptides (ordinate) and the normalized absorbances of the M-protein (abscissa) probed for IgG antibodies. Dotted lines indicate the cutoffs calculated for M4 peptide (0.27), M204 peptide (0.51), and M-protein (29.1%).

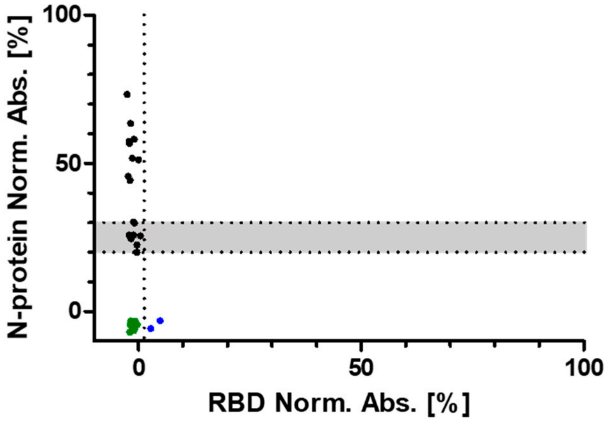

Figure S14. Distribution of the normalized absorbances obtained from ten negative human serum samples (green), two false positive samples identified in the RBD-protein ELISA (blue), and 18 false positive samples in the N-protein ELISA (black) testing each sample for IgG antibodies in ELISA against N-protein expressed in *E. coli* and RBD-protein expressed in HEK cells based IgG ELISA normalized to each. OD_{450}-values were normalized to positive (100%) and negative pools (0%). The cutoff range of the N-protein is indicated as a grey area between dotted lines (20-30%) and the cutoff value of the RBD protein as dotted line (1.25%).

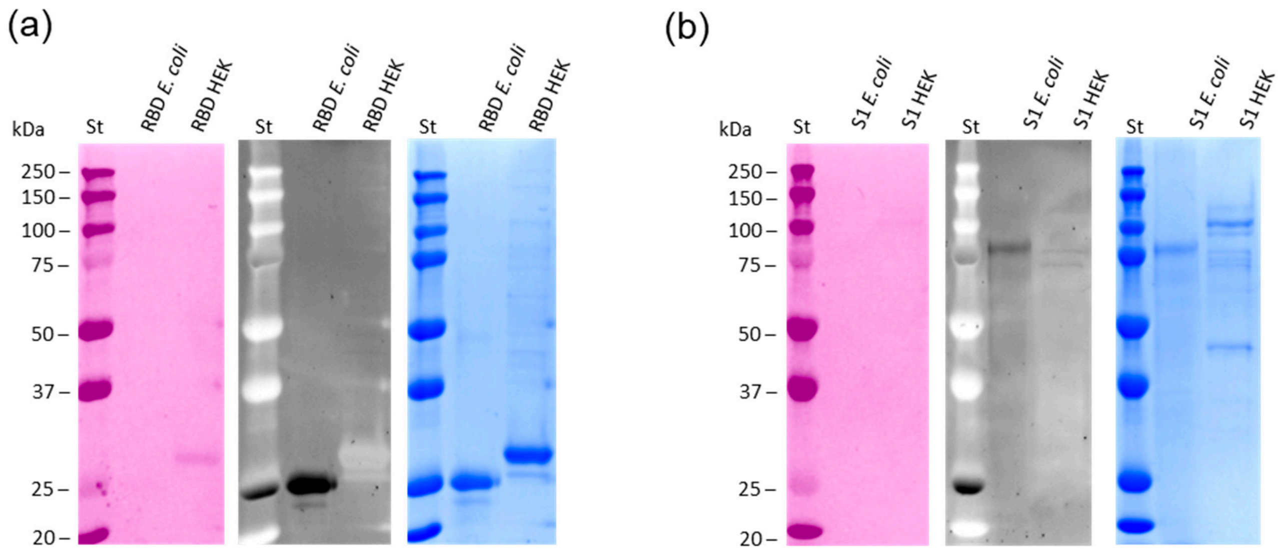

Figure S15. Glyco- (pink), Oriole- (black), and Coomassie-stains (blue) of RBD- (a) and S1-proteins (b) expressed in *E. coli* and HEK. The pink (left) or white (middle) bands in lane 1 resemble glycosylated proteins, while the black band (middle) indicates a non-glycosylated protein, which are all visible in the Coomassie stain.

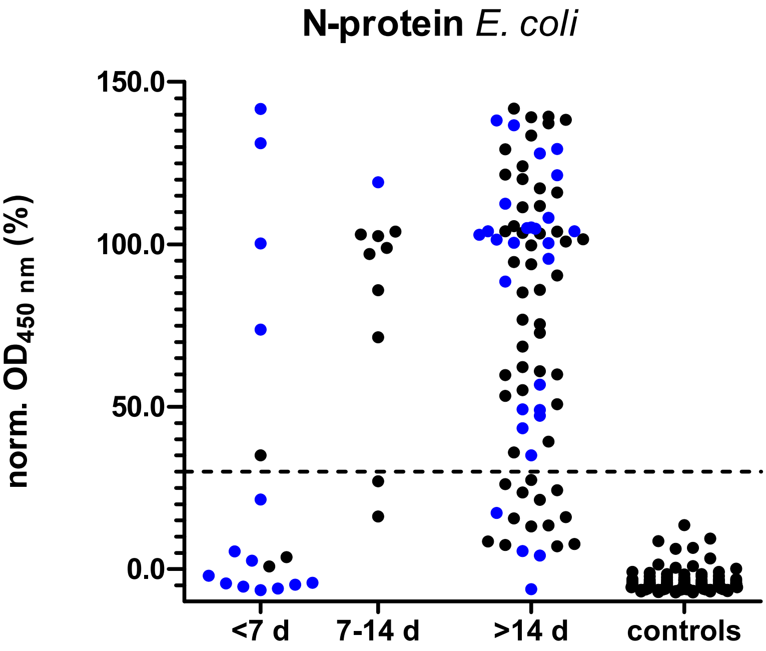

Figure S16. Distribution of normalized OD_{450}-values measured in ELISA for all SARS-CoV-2 positive samples of groups 1 to 3 (N = 113) and 94 control human serum samples coating N-protein expressed in *E. coli* cells and probing for IgG antibodies. Samples were grouped based on the time period after symptom onset (black dots, N = 66) or a positive PCR test (blue dots, N = 47). The OD_{450}-values determined for positive (= 100%) and negative pools (= 0%) on each plate were used for normalization. The cutoff value was obtained from earlier studies and is indicated as dashed line.
